# Ultrasound-assisted extraction of *Peucedanum ostruthium* leaves: a feasible alternative to rhizomes for industrial applications

**DOI:** 10.3389/fphar.2025.1636312

**Published:** 2025-08-12

**Authors:** Chih-Yu Chen, Guey-Horng Wang, Jong-Tar Kuo, Yueh-Te Lin, Hsiu-Ju Huang, Yu-Chi Chang, Ying-Chien Chung

**Affiliations:** ^1^ Department of Tourism and Leisure, Hsing Wu University, New Taipei City, Taiwan; ^2^ Research Center of Natural Cosmeceuticals Engineering, Xiamen Medical College, Xiamen, China; ^3^ Department of Biological Science and Technology, China University of Science and Technology, Taipei City, Taiwan; ^4^ Department of Nutrition and Health Sciences, Kainan University, Taoyuan City, Taiwan; ^5^ Cancer Genome Research Center, Chang Gung Memorial Hospital at Linkou, Taoyuan, Taiwan

**Keywords:** antioxidant activity, cytotoxicity, green technology, *Peucedanum ostruthium*, rhizome extracts

## Abstract

This study investigated the feasibility of using *Peucedanum ostruthium* leaves, typically regarded as waste, as a substitute for the widely used rhizome. To identify the optimal parameters for ultrasound-assisted extraction of *P. ostruthium* leaves, we used Box–Behnken design in combination with response surface methodology. The optimal parameters were as follows: ethanol, 80%; extraction temperature, 61.0°C; ultrasonication duration, 35.5 min; liquid-to-solid ratio, 20; and ultrasonic power, 249 W. The half-maximal inhibitory concentration for the inhibition of extracellular tyrosinase activity, scavenging of free radicals, and suppression of various aging-related and inflammatory enzymes by *P. ostruthium* leaf extract (POLE) were 62.30, 20.62, 8.63, 40.82–91.23, and 12.8–126.4 mg/L, respectively. The minimum bactericidal concentration and minimum fungicidal concentration of POLE for the microorganisms tested in this study were 150–350 and 400–500 mg/L, respectively. Therefore, POLE exhibited strong whitening, antiaging, anti-inflammatory, and antimicrobial properties, surpassing those of the rhizome extract. Furthermore, POLE was biologically safe at concentrations of ≤500 mg/L. The extract exhibited remarkable wound healing capacity (92.5%) at 25 mg/L. The average efficiency of transdermal absorption and penetration reached 98%. A total of 34 predominant volatile organic compounds and 19 major nonvolatile organic compounds were identified in POLE. Molecular docking analysis attributed POLE’s whitening effects to both phenolic acids and flavonoids and its antiaging effects to mainly flavonoids. Furthermore, POLE’s anti-inflammatory and antimicrobial effects were attributed to flavonoids and coumarins. Overall, as a viable alternative to imported *P. ostruthium* rhizome extract, POLE holds promise for applications in cosmeceutical, healthcare, and medical products.

## 1 Introduction

*Peucedanum ostruthium* (L.) Koch, also known as *Imperatoria ostruthium* or masterwort, is a perennial herbaceous flowering plant belonging to the family Apiaceae. It is commonly found in mountainous regions of central and southern Europe, typically growing at altitudes ranging from 800 to 2000 m (Fremstad, 2004). Because of the strong pharmacological activity of *P. ostruthium*, particularly its rhizome, this plant was named the “divine remedy” in the 19th century. Ethnobotanical surveys have indicated that *P. ostruthium* leaves were traditionally used to rapidly treat superficial wounds, which earned it the nickname “magic grass” (Coulter and Rose, 1990). Consequently, the plant has been widely introduced and cultivated outside its native range as a medicinal resource and is now distributed globally (McCardell et al., 2016).

The rhizome extract of *P. ostruthium* possesses antioxidant, anti-inflammatory, anti-allergic, and antibacterial properties (Vogl et al., 2013; Sarkhail, 2014; Mottaghipisheh, 2021). Thus, it has potential applications in medicine, health supplements, and beauty products. However, harvesting the rhizome requires uprooting the entire plant, which is an unsustainable practice. Recently, Danna et al. (2022) reported that 80% ethanol extract of *P. ostruthium* leaves exhibited stronger antioxidant and anti-inflammatory properties than did the rhizome extract. Therefore, *P. ostruthium* leaves, which are regarded as waste products, can be converted into highly functional substances with diverse physiological functions, reducing both waste production and raw material costs. The pharmacological efficacy of *P. ostruthium* extract depends on cultivation conditions, plant part used, and extraction method (McCardell et al., 2016; Vitalini et al., 2021). When both leaf and rhizome of *P. ostruthium* were subjected to 80% ethanol extraction for 6 h, the leaf extract exerted 4.25–6.33 times greater 2,2-diphenyl-1-picrylhydrazyl (DPPH) and 2,2′-azino-bis(3-ethylbenzothiazoline-6-sulfonic acid) (ABTS) radical scavenging activities than did the rhizome extract (Danna et al., 2022). Antioxidant compounds in medicinal plant extracts may exert whitening effects (Chen et al., 2022). Bhanwala et al. (2025) reported the therapeutic potential of phytoconstituents derived from *P*. *ostruthium* as anti-tubercular agents. Zeng et al. (2020) demonstrated that isoimperatorin, the primary component of *P. ostruthium* extract, reduced cellular melanin levels through the microRNA-3619/cystatin B and microRNA-3619/cystatin D pathways. Likewise, Kim and Hyun (2022) indicated that imperatorin, another major component of *P. ostruthium* extract, regulated melanin production through the protein kinase A/cyclic adenosine monophosphate response element-binding protein, extracellular signal-regulated kinase, protein kinase B, and glycogen synthase kinase 3β/β-catenin pathways, thereby exerting whitening effects.

Hiermann and Schanti (1998) reported that a 10% ethanolic extract of *P. ostruthium* rhizome exhibited anti-inflammatory and antipyretic activities following oral administration in animal models. Subsequent research attributed these activities to coumarins, particularly imperatorin and isoprecumin. These substances stimulated the expression of neuronal nitric oxide (NO) synthase in rat PC12 cells and inhibited the activation of nuclear factor-κB and mitogen-activated protein pathways, thereby exerting anti-inflammatory effects (Lammel et al., 2020; Lin et al., 2022). Danna et al. (2022) prepared 80% ethanol extracts from both the rhizome and leaves of *P. ostruthium*. The researchers noted that the leaf extract (concentration: 150 mg/L) inhibited cyclooxygenase activity by 67.3% and lipoxygenase activity by 52% *in vitro*, whereas the rhizome extract inhibited only lipoxygenase activity. These findings confirm that *P. ostruthium* leaf extract (POLE) exerts a stronger anti-inflammatory effect than does the rhizome extract.

Widelski et al. (2018) demonstrated that plants from the genus *Peucedanum* exhibit antibacterial activity against various human pathogens, highlighting their potential for application in medicine. The minimum inhibitory concentration of crude *P. ostruthium* rhizome extract, prepared using hexane or 96% ethanol, was 31.25 mg/L against *Mycobacterium smegmatis*. The extract also exhibited antibacterial activity against *Mycobacterium fortuitum*, *Mycobacterium aurum*, and *Mycobacterium phlei* (Šimunović et al., 2021). When ethyl acetate was used as the extraction solvent, the extract exhibited strong antibacterial activity against *Bacillus cereus*, but not *Escherichia coli* or *Staphylococcus aureus* (Gokay et al., 2010). These findings underscore the importance of selecting appropriate extraction solvents and techniques to enhance the bioactivity of *P. ostruthium* extracts.

In addition to their pharmacological activity, the biosafety of *P. ostruthium* extracts must also be assessed. Abraham et al. (2010) indicated that *P. ostruthium* rhizome extract contains high levels of coumarins and furanocoumarins, which may cause phototoxicity, mutagenicity, carcinogenicity, and hepatotoxicity. However, POLE, which contains low levels of coumarin and its derivatives, represents a safe alternative. Research revealed that POLE at a concentration of 15 mg/L effectively promoted wound closure, highlighting its potential for treating superficial injuries (Danna et al., 2022).

To enhance the physiological activity of plant extracts while reducing toxicity and operational costs, researchers have developed various extraction techniques, such as solvent extraction, water distillation, and steam distillation, supercritical fluid extraction, ultrasound-assisted extraction (UAE), microwave-assisted extraction, pressurized water extraction, pulsed electric field extraction, and high-voltage discharge (Anticona et al., 2020). However, the extraction of *P. ostruthium* components still relies on traditional methods. The use of water–ethanol mixtures as solvents improves activity and extraction efficiency (Palmioli et al., 2019). However, few advanced technologies are available for efficient extraction of *P. ostruthium* components, particularly leaves. Developing simple, cost-effective, and efficient extraction methods is essential for maximizing the value of *P. ostruthium* leaves. UAE has been demonstrated to shorten extraction time, reduce energy consumption, and increase extraction efficiency (Chemat et al., 2017; Wang et al., 2023). Moreover, it yields higher levels of active compounds than does conventional methods (Chemat et al., 2017; Wang et al., 2023). Thus, the primary objective of this study was to utilize UAE to extract bioactive compounds from *P. ostruthium* leaves and evaluate their tyrosinase inhibitory, antioxidant, antimicrobial, anti-inflammatory, and antiwrinkle activities. Subsequently, the mechanisms underlying the physiological activity of the extracts were elucidated by determining their compositions and bioactive contents (levels of phenolic acids, flavonoids, triterpenes, phenylethanoid glycosides, and other compounds). Additionally, molecular docking analysis was performed to investigate the binding interactions between POLE’s chemical constituents and corresponding enzymatic targets.

## 2 Materials and methods

### 2.1 Preparation of raw materials and extracts

Three commercially available 100% pure *P. ostruthium* rhizome powders from Switzerland, China, and Italy—designated as rhizome extract-1, rhizome extract-2, and rhizome extract-3, respectively—were used as controls. Fresh fallen leaves of *P. ostruthium* were collected from Sanxing Township Farm in Yilan County, Taiwan. For UAE, the leaves were shade-dried for 3 days, crushed, and passed through a 4-mm sieve.

The operating conditions for single-factor UAE were as follows: extraction solvent, water or hexane or 80% ethanol; extraction temperature, 30°C–80°C; ultrasonication duration, 10–60 min; liquid-to-solid (L/S) ratio, 15–40 mL/g; and ultrasonic power, 150–350 W. These conditions were tested to determine key parameters for optimal experimental design. Ultrasonic extraction was performed using the PS-60AL ultrasonic machine (capacity: 15 L; frequency: 40 kHz) (Shenzhen Kejie Ultrasonic Technology Co., Shenzhen, China). First, the L/S ratio was set to 20 mL/g by mixing 10 g of leaf powder with 200 mL of extraction solvent in an Erlenmeyer flask. Then, the operating parameters were adjusted one at a time to evaluate extraction efficiency. Notably, extraction efficiency was estimated in terms of total phenolic content (TPC) and total flavonoid content (TFC), given that phenolic acids and flavonoids are major bioactive constituents of *P. ostruthium* extracts (Sarkhail, 2014).

### 2.2 UAE

To explore the interactions between various parameters and optimize production processes, we combined Box–Behnken design (BBD) with response surface methodology (RSM), thereby determining optimal operating parameters and deriving simulation equations. On the basis of BBD, 29 sets of experiments were conducted, comprising 24 unique operating parameters across three coded levels (−1, 0, 1) and five replicates at the center point (0, 0, 0). To model the correlations of the four parameters with TPC and TFC, a second-order polynomial equation was constructed using Design-Expert software (version 12). Analysis of variance (ANOVA) was performed to determine the significance of the effect of each parameter on TPC and TFC (*p* values) as well as the model fit (*F*-value and *R* ^2^). Extracts obtained under the optimal conditions were freeze-dried for the analysis of pharmacological activity.

### 2.3 Measurement of tyrosinase activity and cellular melanin content

To measure extracellular tyrosinase activity, lyophilized POLE (powder) was dissolved in 1 g/L dimethyl sulfoxide and diluted to various concentrations. Then, 30 μL of POLE was sequentially mixed with 970 μL of phosphate-buffered saline (PBS) (0.05 mM, pH 6.8), 1 mL of tyrosine solution (100 mg/L), and 1 mL of tyrosinase solution (activity: 350 U/mL). After 20-min mixing, absorbance was measured at 490 nm by using a ultraviolet–visible (UV–vis) spectrophotometer (Thermo Fisher Scientific, Waltham, MA, United States) (Wang et al., 2017).

Intracellular tyrosinase activity was measured using human epidermal melanocytes (HEMns) (Cascade cat. C-102-5C, Cascade Biologics, Inc., Portland, OR, United States). In brief, HEMns were seeded in 24-well plates (density: 5 × 10^6^ cells/well) containing Medium 254 supplemented with Human Melanocyte Growth Supplement (HMGS) and incubated at 37°C under 5% CO_2_. After 24 h, varying concentrations of POLE were added and incubated for another 24 h. Then, the cells were washed with PBS, treated with trypsin, subjected to ultrasonic vibration, and centrifuged. The supernatant was mixed with 2.5 mM levodopa in a 96-well plate and incubated for 60 min. Absorbance was measured at 475 nm by using an Epoch ELISA reader (BioTek Instruments, Santa Clara, CA, United States) (Wu et al., 2021).

Cellular melanin content was measured using a method similar to the aforementioned one. Melanin-containing pellets were obtained through centrifugation and dissolved in a solution of 1 N NaOH and 10% dimethyl sulfoxide. The mixture was incubated at 70°C for 1.5 h. Melanin content in HEMns was determined by measuring absorbance at 450 nm. Concentrations were estimated from a calibration curve correlating OD_405_ with melanin concentration (Wu et al., 2021).

### 2.4 Assessment of antioxidant properties

TPC was determined as follows: 500 μL of POLE at various concentrations was mixed with 1 mL of 1 N Folin–Ciocalteu phenol reagent and 1 mL of 7.5% Na_2_CO_3_ solution. The mixture was allowed to stand for 3 h during the single-factor experiment. Then, the solution was centrifuged at 3,000 rpm for 15 min. Absorbance of the supernatant was measured at 760 nm (OD_760_) by using a UV–vis spectrophotometer (Kujala et al., 2000; Chen et al., 2025). OD_760_ indicated the relative phenolic content in the sample. The lyophilized extract obtained under optimal conditions was diluted to various concentrations, and corresponding OD_760_ values were estimated using the aforementioned method. TPC was estimated from a calibration curve of OD_760_ against TPC (expressed as milligrams of gallic acid equivalent per gram of dry weight (mg-GAE/g-dw) (Kujala et al., 2000).

TFC was measured using the aluminum chloride colorimetric method (Pourmorad et al., 2006; Chen et al., 2025). In the single-factor experiment, 600 μL of POLE at various concentrations was mixed with 600 μL of 2% AlCl_3_ solution and incubated at room temperature for 60 min. Then, absorbance was measured at 420 nm (OD_420_) by using a UV–vis spectrophotometer. OD_420_ indicated the relative flavonoid content in the sample. The lyophilized extract obtained under optimal conditions was diluted to various concentrations, and corresponding OD_420_ values were estimated using the aforementioned method. TFC was calculated using the calibration curve between OD_420_ and TFC (expressed as milligrams of rutin equivalents per gram of dry weight mg-RE/g-dw) (Pourmorad et al., 2006).

To measure DPPH free radical scavenging activity, 2 mL of POLE at various concentrations was mixed with 0.5 mL of 0.25 mM DPPH solution. After 30-min incubation in the dark, absorbance was measured at 517 nm by using a UV–vis spectrophotometer (Wu et al., 2018). A sample without POLE was used as the control. The DPPH radical scavenging activity (%) was calculated using Equation 1:

$$\text{DPPH scavenging activity }\left(\%\right)=\left\{1‐\frac{\left({\mathrm{OD}}_{517}\;\mathrm{of}\;\mathrm{POLE}\right)}{{\mathrm{OD}}_{517}\;\mathrm{of}\;\mathrm{control}}\right\}\times 100$$ (1)

Butylated hydroxytoluene (BHT) was used as a positive control in both DPPH and ABTS radical scavenging assays.

To determine ABTS free radical scavenging activity, equal volumes of 2.45 mM K_2_S_2_O_8_ solution and 7 mM ABTS solution were mixed. After 16 h of incubation in the dark, the mixture was diluted until its absorbance at 734 nm reached 0.7. Then, 20 μL of POLE at various concentrations was mixed with 180 μL of the ABTS radical solution. After 30-min incubation, absorbance was measured at 734 nm by using an ELISA plate reader (Wu et al., 2018). A sample without POLE was used as the control. The ABTS radical scavenging activity (%) was calculated using Equation 2:

$$\text{ABTS scavenging activity }\left(\%\right)=\left\{1‐\frac{\left({\mathrm{OD}}_{734}\;\mathrm{of}\;\mathrm{POLE}\right)}{{\mathrm{OD}}_{734}\;\mathrm{of}\;\mathrm{control}}\right\}\times 100$$ (2)

### 2.5 Measurement of antiaging activity

We measured intracellular activity of matrix metalloproteinase-1 (MMP-1) and extracellular activities of collagenase, elastase, and hyaluronidase. MMP-1 activity was measured as follows. First, CCD966SK cells (Biological Resource Preservation and Research Center (BCRC) in Taiwan) were seeded into 24-well plates (density: 1.0 × 10^5^ cells/well) containing Minimum Essential Medium (MEM) supplemented with 10% fetal bovine serum (FBS) and incubated at 37°C under 5% CO_2_. After 24 h, the medium was discarded. Then, 200 μL of POLE was added and incubated for 48 h (Yoshikawa et al., 1987; Chen et al., 2022). Subsequently, MMP-1 activity was determined using a commercial human MMP-1 ELISA kit (RayBiotech, Norcross, GA, United States), following the manufacturer’s instructions.

Collagenase activity was measured using the fluorescent dye-quenched gelatin method (Vandooren et al., 2011; Chen et al., 2022). In brief, 20 μL of POLE at various concentrations, 100 μL of collagenase (activity: 1 U/mL), and 15 μg/mL dye-quenched gelatin were mixed in a 96-well plate and incubated for 15 min. Absorbance was measured at an excitation wavelength of 485 nm and an emission wavelength of 528 nm by using a Synergy two microplate reader (BioTek Instruments, Santa Clara, CA, United States). Elastase activity was measured following a method described by Wu et al. (2018). For this, 20 μL of POLE, 50 μL of PBS, and 50 μL of neutrophil elastase (activity: 0.3 U/mL) were added sequentially to a 48-well plate and incubated at 37°C for 10 min. Then, 5 μL of the substrate methoxysuccinyl-Ala-Ala-Pro-Val-p-nitroanilide was added, and the mixture was incubated for another 10 min. Absorbance was measured at 405 nm by using an ELISA plate reader. Hyaluronidase activity was measured using the method of Thring et al. (2009). In brief, 100 μL of POLE, 100 μL of hyaluronidase (3,000 U/mL), and 500 μL of hyaluronic acid solution (5,000 mg/L) were mixed and incubated at 37°C for 40 min. Then, 2 mL of *p*-dimethylaminobenzaldehyde was added. Absorbance was measured at 570 nm by using a UV–vis spectrophotometer (Thring et al., 2009). Epigallocatechin gallate (EGCG) was used as a positive control for MMP-1, collagenase, and hyaluronidase; 1,10-phenanthroline and gallic acid were used as positive controls for elastase; and ursolic acid was used as an additional positive control for hyaluronidase.

### 2.6 Measurement of antimicrobial activity

To measure the antimicrobial activity of POLE, five standard strains—*S. aureus* ATCC 6538, *E. coli* ATCC 8739, *Pseudomonas aeruginosa* ATCC 9027, *Candida albicans* ATCC 10231, and *Aspergillus brasiliensis* ATCC 16404—were used in accordance with the United States Pharmacopeia 51 guidelines for antimicrobial efficacy testing (USP <51>). Bacterial strains were cultured at 32.5°C in Tryptic Soy Broth, whereas fungal strains were cultured at 22.5°C in Sabouraud Dextrose Broth. After incubation, 1 mL of POLE at various concentrations, 1 mL of bacterial suspension (5.0 × 10^6^ CFU/mL), and 8 mL of fresh Tryptic Soy Broth were added to a test tube and incubated at 32.5°C for 24 h. Minimum bactericidal concentration (MBC) was determined using standard serial dilution methods in accordance with the Clinical Laboratory Standards Institute (CLSI) guidelines (2018). Similarly, to evaluate antifungal activity, 1 mL of POLE at various concentrations, 1 mL of *Aspergillus brasiliensis* spore suspension (6.0 × 10^5^ spores/mL) or *C. albicans* yeast suspension (6.0 × 10^5^ CFU/mL), and 8 mL of fresh Sabouraud Dextrose Broth were added to a test tube and incubated at 22.5°C for 5*–*7 days. The minimum fungicidal concentration (MFC) was determined using standard serial dilution methods. Subsequently, a lotion was formulated considering the MBC and MFC values of POLE, and its antimicrobial effects were assessed on days 14 and 28 of treatment.

### 2.7 Measurement of anti-inflammatory activity

The intracellular levels of inflammatory factors such as NO, interleukin (IL)-6, and tumor necrosis factor (TNF)-α were determined (Huang et al., 2019). Moreover, the extracellular activities of bovine serum albumin (BSA), lipoxygenase (LOX), and cyclooxygenase-2 (COX-2) were measured (Smeriglio et al., 2020). In brief, Raw264.7 cells (6 × 10^5^ cells/mL) (obtained from BCRC) were cultured in the presence of lipopolysaccharide (1 mg/L) and various concentrations of POLE. Two control groups were included: one group comprised Raw264.7 cells cultured in Dulbecco’s Modified Eagle Medium (DMEM) alone, whereas the other group comprised Raw264.7 cells cultured with lipopolysaccharide but without POLE. After 24-h incubation, equal volumes of cell suspension and Griess reagent were mixed and allowed to react for 10 min. Absorbance was measured at 550 nm by using an ELISA plate reader. The level of NO produced by Raw264.7 cells was estimated from a standard curve constructed using NaNO_2_. Concurrently, the levels of IL-6 and TNF-α were measured using the IL-6 Quantikine ELISA Kit and TNF-α Quantikine ELISA Kit, respectively (R&D Systems, Minneapolis, MN, United States).

The ability of POLE to inhibit the denaturation of BSA was assessed by mixing 100 µL of 0.4% BSA solution, 20 µL of PBS (pH 5.3), and 80 µL of POLE in a 48-well plate and incubating at 70°C for 30 min. Postincubation changes in absorbance at 595 nm were analyzed to evaluate the inhibitory effect of POLE on of BSA denaturation. The activities of LOX and COX-2 were measured using the Lipoxygenase Inhibitor Screening Assay Kit and COX Inhibitor Screening Assay Kit, respectively (Cayman Chemical, Ann Arbor, MI, United States).

### 2.8 Assessment of cell viability and wound healing capacity

The viability of epidermal (HaCaT) cells, HEMns, dermal (CCD966SK) cells, and Raw264.7 cells was assessed using the MTT assay (Wang et al., 2019). First, the cells were seeded in 48-well plates (density of 3 × 10^5^ cells/well) and incubated at 37°C under 5% CO_2_. After 24 h, varying concentrations of POLE or fresh medium (control) were added and incubated for 72 h. The medium compositions for various cells were as follows: DMEM supplemented with 10% FBS and 1% streptomycin–penicillin for HaCaT cells, Medium 254 supplemented with HMGS for HEMns, MEM supplemented with 10% FBS for CCD966SK cells, and DMEM alone for Raw264.7 cells. After incubation, cell viability was assessed using the MTT Cell Growth Assay Kit (Sigma-Aldrich, St. Louis, MO, United States), following the manufacturer’s instructions.

An *in vitro* wound healing assay was performed following the method of Bazzicalupo et al. (2021). In brief, HaCaT and CCD966SK cells were seeded in 12-well plates (density: 2 × 10^5^ cells/well) until a confluent monolayer was formed. Then, this layer was scratched using a sterile 200-μL pipette tip. Next, varying concentrations of POLE were added and incubated for 24 h. After incubation, the cells were fixed using FineFIX solution and stained with toluidine blue O. Wound healing was evaluated by capturing stereomicroscopic images. For HaCaT cells, wound width was measured using ImageJ software (http://rsbweb.nih.gov/ij). For CCD966SK cells, wound closure was quantified by measuring cell density in the wounded area, assessing 50 wounded cells at both 0 and 24 h. Data are presented as mean ± standard deviation values. Allantoin was used as the positive control in the wound healing assay.

### 2.9 Analysis of chemical composition

Evidence suggests that key bioactive compounds or secondary metabolites present in *P. ostruthium* extract include coumarins, phenolic acids, flavonoids, and terpenoids (Cisowski et al., 2001; Joa et al., 2011; Vitalini et al., 2021; Garzoli et al., 2022). Thus, we performed both gas chromatography (GC) and liquid chromatography (LC) to analyze the chemical constituents of POLE. GC–mass spectrometry (MS) was performed using a PerkinElmer Clarus 500 system (Waltham, MA, United States) equipped with a VF-1 capillary column (0.25 mm ID × 30 m). Helium was used as the carrier gas, and the flow rate was 1 mL/min. The oven was first maintained at 60°C for 5 min, then heated to 220°C at a rate of 6°C/min, and then maintained at 220°C for 20 min (Vitalini et al., 2022). The ionization energy for MS was set to 70 eV, and the scanning range was 35*–*400 m/z. The resulting mass spectra were identified by comparing them with the Wiley mass spectral library (version 8.0) and National Institute of Standards and Technology mass spectral library (version 11).

Reverse-phase high-performance LC coupled with electrospray ionization MS (RP-HPLC-ESI-MS) was performed to analyze nonvolatile components of POLE by using an Agilent 1100 series HPLC system equipped with a G1315 diode-array detector and an Agilent 6320 ion trap mass spectrometer (Santa Clara, CA, United States). Chromatographic separation was achieved using a Luna Omega PS C18 column (150 × 2.1 mm, 5 µm). The mobile phase comprised solvent A (0.1% formic acid in water) and solvent B (methanol). The gradient elution was programmed as follows: 0*–*3 min, 0% B; 3*–*9 min, 3% B; 9*–*24 min, 12% B; 24–30 min, 20% B; 30–33 min, 25% B; 33–43 min, 30% B; 43–63 min, 50% B; 66–76 min, 60% B; 76–81 min, 60% B; and 81–86 min, 0% B, followed by equilibration for 4 min. Spectral data were collected in the 190–600 nm range, with specific recordings obtained at 220, 260, 292, 330, and 370 nm. The ion source was operated at the following settings: capillary voltage, 3.5 kV; nitrogen pressure, 40 psi; gas temperature, 350°C; gas flow rate, 9 L/min; and skimmer voltage, 40 V. The scanning range was 90–1,000 m/z in the full scan mode (Smeriglio et al., 2020).

### 2.10 Molecular simulation and potential inhibitory mechanisms

To investigate the correlations between various POLE components and enzyme inhibition and to elucidate the potential mechanisms underlying each component’s physiological activity, we performed molecular docking analysis. Major compounds in POLE were identified on the basis of chemical composition analysis. Target enzymes included those associated with melanogenesis (tyrosinase: PDB code 5M8L), aging (MMP-1: PDB code 3SHI, elastase: PDB code 1FLE, collagenase: PDB code 4FVL, and hyaluronidase: PDB code 2PE4), antimicrobial activity (tyrosyl-tRNA synthetase (TyrRS: PDB code 2TS1) and sterol 14α-demethylase (CYP51: PDB code 5TZ1), and inflammation (LOX: PDB code 4NRE and COX-2: PDB code 5KIR). The analyses clarified the mechanisms underlying POLE’s whitening, antimicrobial, antiaging, and anti-inflammatory activities. Information on the three-dimensional crystal structures of target proteins was obtained from the Protein Data Bank, whereas information on the three-dimensional structures of selected POLE compounds was obtained from the PubChem database. Furthermore, we utilized UniProt database to obtain target protein information, including protein sequences and annotations. Flexible molecular docking was performed using AutoDockTools (version 1.5.7) and AutoDock Vina program to evaluate receptor–ligand binding affinities (Yuan et al., 2017). The optimal binding pose was identified on the basis of the lowest receptor–ligand binding energy. Additionally, the center size of docking box for targeted docking ranged from 20 Å × 20 Å × 20Å to 30 Å × 30 Å × 30Å.

### 2.11 Evaluation of transdermal absorption and permeation efficiency

The Phoenix DB-6 Diffusion Testing System (Teledyne Technologies, Chatsworth, CA, United States) was used in this study. POLE (400 mg/L) or a lotion containing 400 mg/L POLE was introduced into the donor chamber of the aforementioned system, whereas physiological saline was introduced into the receptor chamber. A Strat-M membrane (Merck Millipore, Billerica, MA, United States) was positioned between the donor and receptor chambers to simulate skin permeability. The diffusion cells were maintained in a 32°C water bath. The system automatically collected samples every 15 min, and the same volume of physiological saline was replenished after each sampling. The permeation efficiency plateaued after 2 h for POLE and after 3 h for the lotion. Thus, the TPC and TFC of the samples in the donor chamber, Strat-M membrane, and receptor chamber were analyzed at the 2- and 3-h time points, respectively, for assessments of transdermal absorption and permeation efficiency (Kichou et al., 2023).

### 2.12 Statistical analysis

Data were analyzed using one-way ANOVA, followed by Duncan’s multiple range test, and are presented as the means ± standard deviations of at least three replicates. Statistical analyses were performed using SPSS (version 20.0; IBM, Armonk, NY, United States). A p value of <0.05 was considered statistically significant.

## 3 Results and discussion

### 3.1 Single-factor experiment

**FIGURE 1:**
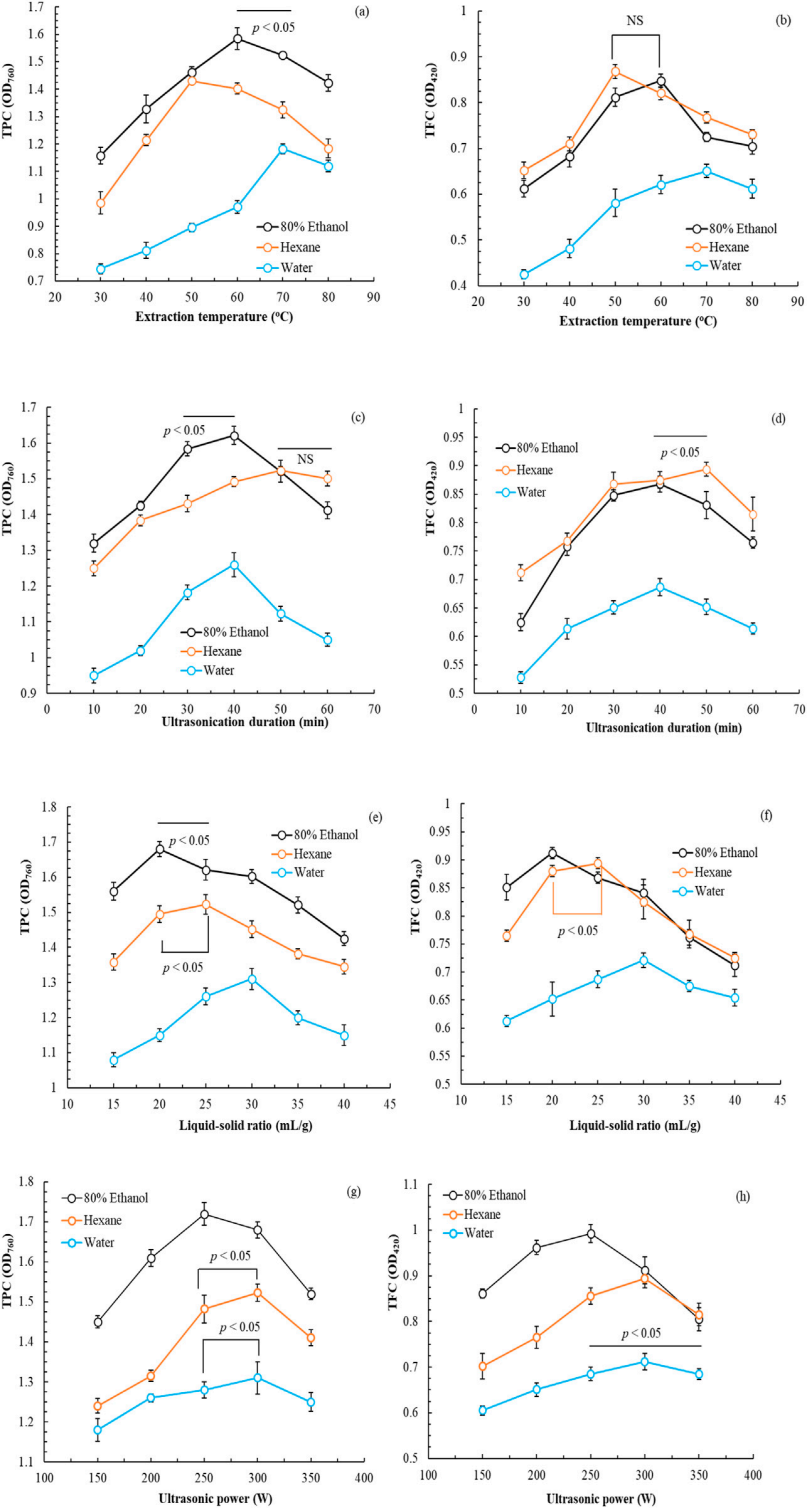
Effects of extraction temperature **(a,b)**, ultrasonication duration **(c,d)**, liquid-to-solid ratio **(e,f)**, and ultrasonic power **(g,h)** on the total phenolic and flavonoid contents of *Peucedanum ostruthium* leaf extract. Data are presented as the means and standard deviations of three independent experiments (NS, nonsignificant; *p* > 0.05, Significance, *p < 0.05).

Figure 1 depicts the effects of various parameters (extraction temperature, ultrasonication duration, L/S ratio, and ultrasonic power) on the TPC and TFC of POLE. Similar trends were observed for all variables, where increases in extraction temperature, ultrasonication duration, L/S ratio, and ultrasonic power initially increased TPC and TFC production; however, TPC and TFC decreased with time. In the single-factor experiment, the optimal parameters when 80% ethanol was used as the solvent were as follows: extraction temperature, 60°C; ultrasonication duration, 40 min; L/S ratio, 20 mL/g; and ultrasonic power, 250 W. When hexane was used as the solvent, the optimal parameters were as follows: extraction temperature, 50°C; ultrasonication duration, 50 min; L/S ratio, 25 mL/g; and ultrasonic power, 300 W. When water was used as the solvent, the optimal parameters were as follows: extraction temperature, 70°C; ultrasonication duration, 40 min; L/S ratio, 30 mL/g; and ultrasonic power, 300 W.

In summary, the use of 80% ethanol ensured the highest TPC and TFC of POLE. This finding aligns with that of Palmioli et al. (2019), who indicated that aqueous alcohol enhances extract activity. The optimal conditions were as follows: extraction temperature, 60°C; ultrasonication duration, 40 min; L/S ratio, 20 mL/g; and ultrasonic power, 250 W. Notably, these conditions not only optimized the TPC and TFC but also minimized energy consumption due to the reduced extraction power. Under these conditions, the OD_760_ value for TPC was 1.721 ± 0.028, whereas the OD_420_ value for TFC was 0.992 ± 0.020.

### 3.2 Optimization of UAE parameters

On the basis of the results of the single-factor experiment, we used BBD plus RSM to identify the optimal operating parameters and corresponding simulation equations. The central parameters were as follows: extraction solvent, 80% ethanol; extraction temperature, 60°C (*x*_1_); ultrasonication duration, 40 min (*x*_2_); L/S ratio, 20 mL/g (*x*_3_); and ultrasonic power, 250 W (*x*_4_). Multiple regression analysis was performed to develop simulation equations for TPC and TFC. The Equations 3 and 4 are as follows:

$$\text{TPC }\left({\text{OD}}_{760}\right):y=-10.03+0.2116x_{1}+0.0510x_{2}+0.148x_{3}+0.0229x_{4}\;-\;0.002047x_{1}^{2}\;-\;0.000527x_{2}^{2}\;-\;0.005x_{3}^{2}\;-\;0.000054x_{4}^{2}\;-\;0.000185\left(x_{1}\times x_{2}\right)+0.000990\left(x_{1}\times x_{3}\right)+0.000096\;\left(x_{1}\times x_{4}\right)\;-\;0.000040\left(x_{2}\times x_{3}\right)\;-\;0.000002\left(x_{2}\times x_{4}\right)\;-\;0.000043\left(x_{3}\times x_{4}\right)$$ (3)

$$\text{TFC }\left({\text{OD}}_{420}\right):y=-3.865+0.0772x_{1}+0.0217x_{2}+0.0579x_{3}+0.01032x_{4}\;-\;0.000522x_{1}^{2}\;-\;0.000207x_{2}^{2}\;-\;0.002013x_{3}^{2}\;-\;0.000019x_{4}^{2}\;-\;0.000243\left(x_{1}\times x_{2}\right)+0.000005\left(x_{1}\times x_{3}\right)\;-\;0.00002\left(x_{1}\times x_{4}\right)+0.0005\left(x_{2}\times x_{3}\right)\;-\;0.000013\left(x_{2}\times x_{4}\right)+0.000026\left(x_{3}\times x_{4}\right)$$ (4)

In the ANOVA, the *F*-value and *R* ^2^ for the model predicting TPC were 2.62 and 0.9535, respectively, whereas those for the model predicting TFC were 5.98 and 0.9750, respectively. These findings indicate a strong positive correlation between the experimental and predicted values, suggesting the suitability of the models for value prediction. The optimal extraction conditions for TPC were as follows: extraction temperature, 60.70°C; ultrasonication duration, 36.47 min; L/S ratio, 19.55; and ultrasonic power, 257.58 W. By contrast, the optimal extraction conditions for TFC were as follows: extraction temperature, 61.20°C; ultrasonication duration, 35.42 min; L/S ratio, 20.13; and ultrasonic power, 248.40 W. Thus, POLE was prepared under conditions that were close to those ideal for TFC extraction, specifically an extraction temperature of 61.0°C, an ultrasonication duration of 35.5 min, a L/S ratio of 20, and an ultrasonic power of 249 W.

TPC and TFC can be quantified using standard curves for absorption. The equation for TPC is as follows: *y* = 0.00578*x* + 0.04680 (*R* ^2^ = 0.9991). Conversely, the equation for TFC is as follows: *y* = 0.00085*x* − 0.05291 (*R* ^2^ = 0.9982). Thus, the optimal concentrations for TPC and TFC were determined to be 145.82 mg-GAE/g-dw and 637.45 mg-RE/g-dw, respectively. The ratio of UAE-based extraction of *P. ostruthium* leaves was 33.6% ± 2.7%, slightly higher than that when only ethanol extraction was performed (31.7%) (Danna et al., 2022).

### 3.3 Whitening activity of POLE

**FIGURE 2:**
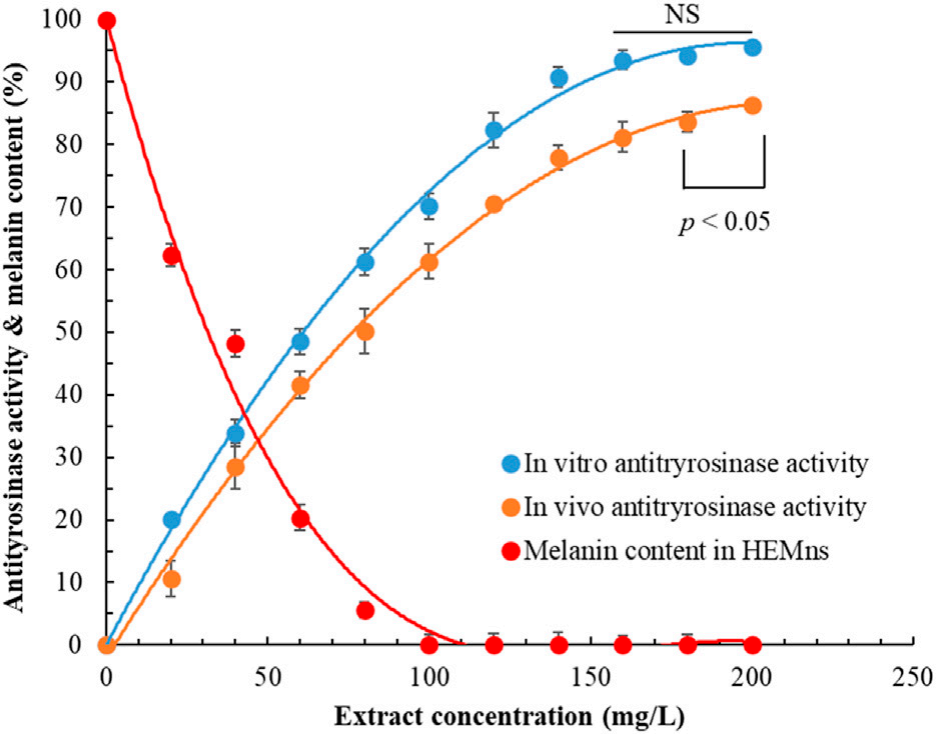
Effects of *Peucedanum ostruthium* leaf extract on extracellular antityrosinase activity and intracellular antityrosinase activity and melanin content. Human epidermal melanocytes were used in the experiment. The ultrasound-assisted extraction operating conditions were as follows: extraction solvent, 80% ethanol; extraction temperature, 61.0°C; ultrasonication duration, 35.5 min; liquid-to-solid ratio, 20 mL/g; and ultrasonic power, 249 W. Data are presented means and standard deviations of three independent experiments (NS, nonsignificant; *p* > 0.05, Significance, *p < 0.05).

Figure 2 presents the effects of POLE at varying concentrations on both extracellular and intracellular antityrosinase activities and melanin content in HEMns. As extract concentration increased, both extracellular and intracellular antityrosinase activities increased, whereas melanin content decreased. Notably, at a POLE concentration of 100 mg/L, the intracellular melanin content reached 0. Regression analysis indicated that the half-maximal inhibitory concentration (IC_50_) values for extracellular and intracellular antityrosinase activities were 62.30 ± 1.25 and 79.21 ± 1.90 mg/L, respectively, implying that POLE exhibited superior inhibitory effects on extracellular tyrosinase than on intracellular tyrosinase. Moreover, the IC_50_ value for the inhibition of intracellular melanin production was 38.60 ± 0.82 mg/L. These results suggest that the mechanism through which POLE inhibits intracellular melanin production is not solely attributable to the inhibition of tyrosinase activity, indicating a complex whitening mechanism that involves the upregulation of tyrosinase, tyrosinase-related protein-1, and tyrosinase-related protein-2 (Kim and Hyun, 2022).

### 3.4 Antioxidant properties of POLE

Table 1 presents the diverse antioxidant properties of POLE. The results indicated that the IC_50_ values for the scavenging of DPPH and ABTS free radicals by POLE were 20.62 ± 0.85 and 8.63 ± 0.27 mg/L, respectively. The corresponding values for BHT the positive control, were 53.51 ± 1.54 and 42.86 ± 1.75 mg/L, respectively. The IC_50_ values of three commercial rhizome extracts for scavenging DPPH radicals ranged from 62.41 ± 1.35 to 127.15 ± 3.41 mg/L, whereas those for scavenging ABTS radicals ranged from 14.60 ± 0.95 to 48.62 ± 1.08 mg/L. These results indicate that the antioxidant activity of POLE is significantly superior to that of the positive control and commercial rhizome extracts. Thus, *P. ostruthium* leaves hold great potential for commercial applications. Notably, the activity of POLE in scavenging ABTS radicals was 2.38 times greater than that in scavenging DPPH radicals, suggesting that most of the active compounds in this extract are hydrophilic. Similar findings were reported for POLE obtained through hydrodistillation (Garzoli et al., 2022). The results presented in Table 1 also indicate that samples with similar polyphenol contents exhibit varying IC_50_ values. It is speculated that, although the total polyphenol content may be comparable, the types and proportions of specific polyphenolic compounds could differ. In other words, if a more active polyphenol is present in higher concentrations in a particular sample, its IC_50_ value would be lower (Danna et al., 2022).

**TABLE 1:** Antioxidant properties of *Peucedanum ostruthium* leaf extract.

Extracts	DPPH (IC_50_) (mg/L)	ABTS (IC_50_) (mg/L)	TPC (mg-GAE/g-dw)	TFC (mg-RE/g-dw)
Ethanolic extract	20.62 ± 0.85^a^	8.63 ± 0.27^a^	145.81 ± 3.61^b^	637.45 ± 7.52^d^
Rhizome extract-1	62.41 ± 1.35^b^	20.81 ± 1.12^c^	147.23 ± 2.14^c^	424.97 ± 5.87^c^
Rhizome extract-2	100.83 ± 2.85^c^	48.62 ± 1.08^d^	142.88 ± 3.95^a^	344.57 ± 1.68^b^
Rhizome extract-3	127.15 ± 3.41^d^	14.60 ± 0.95^b^	154.33 ± 3.21^d^	277.15 ± 3.41^a^

The ultrasound-assisted extraction operating conditions were as follows: extraction solvent, 80% ethanol; extraction temperature, 61.0°C; ultrasonication duration, 35.5 min; liquid-to-solid ratio, 20 mL/g; and ultrasonic power, 249 W. The positive control for the DPPH assay was ascorbic acid, with an IC5_0_ value of 22.78 ± 1.26 mg/L, and for the ABTS assay, it was 8.12 ± 0.34 mg/L. Within each column, different superscript letters (a–d) indicate statistically different values according to Duncan’s test at *p* < 0.05.

The TPC and TFC of POLE were 145.81 ± 3.61 mg-GAE/g-dw and 637.45 ± 7.52 mg-RE/g-dw, respectively. POLE exhibited a significantly higher TFC than did the commercial rhizome extracts. Similarly, POLE exhibited a higher TFC than did the leaf extract treated solely with 80% ethanol for 6 h (529.15 ± 3.85 mg-RE/g-dw) (Danna et al., 2022). These findings suggest that POLE can replace the commonly used rhizome extract. Although the TPC values of POLE and rhizome extracts were similar, their antioxidant activities differed significantly, likely because of variations in their component profiles. Similar findings were reported by Danna et al. (2022), who indicated that the flavonoid content was relatively high in the 80% ethanol leaf extract. The IC_50_ values for DPPH and ABTS radical scavenging by the 80% ethanol leaf extract were 24.11 and 12.14 mg/L, respectively (Danna et al., 2022), which were lower than those obtained with the incorporation of UAE.

### 3.5 Antiaging activity of POLE

Research suggests that POLE holds potential for commercial applications; however, very few studies have explored its antiaging properties. Table 2 presents the IC_50_ values of POLE in relation to antiaging activity. The IC_50_ values of POLE (obtained through UAE) for inhibiting MMP-1, collagenase, elastase, and hyaluronidase were 91.23 ± 2.34, 68.40 ± 1.54, 52.35 ± 1.68, and 40.82 ± 2.74 mg/L, respectively. Notably, the three commercial rhizome extracts exerted no inhibitory effect on MMP-1. Only rhizome extract-1 exerted a slight inhibitory effect on collagenase (IC_50_: 506.40 ± 6.34 mg/L). Rhizome extract-2 and rhizome extract-3 exerted limited inhibitory effects on other antiaging enzymes, with IC_50_ values ranging from 384.20 ± 3.21 to 815.20 ± 9.75 mg/L. The IC_50_ values of EGCG (positive control) for MMP-1, collagenase, and hyaluronidase were 90.63 ± 1.84, 67.26 ± 2.04, and 162.8 ± 2.95 mg/L, respectively. Moreover, the IC_50_ values of 1,10-phenanthroline and gallic acid (positive controls) for elastase were 46.75 ± 1.92 and 132.65 ± 4.01 mg/L, respectively. Furthermore, the IC_50_ value of ursolic acid (positive control) for hyaluronidase was 32.61 ± 0.91 mg/L. These results indicate that although the antiaging activity of POLE is not only significantly superior to that of the rhizome extracts, it is similar to that of commonly used commercial chemical drugs.

**TABLE 2:** Antiaging activity (IC_50_, mg/L) of *Peucedanum ostruthium* leaf extract.

Extracts	MMP-1	Collagenase	Elastase	Hyaluronidase
Ethanolic extract	91.23 ± 2.34	68.40 ± 1.54^a^	52.35 ± 1.68^a^	40.82 ± 2.74^a^
Rhizome extract-1	−^*^	506.40 ± 6.34^b^	384.20 ± 3.21^b^	627.80 ± 4.68^b^
Rhizome extract-2	−	−	−	815.20 ± 9.75^c^
Rhizome extract-3	−	−	645.80 ± 3.49^c^	−

The ultrasound-assisted extraction operating conditions were as follows: extraction solvent, 80% ethanol; extraction temperature, 61.0°C; ultrasonication duration, 35.5 min; liquid-to-solid ratio, 20 mL/g; and ultrasonic power, 249 W. Within each column, different superscript letters (a–c) indicate statistically different values according to Duncan’s test at *p* < 0.05. *IC_50_ > 1,500 mg/L.

### 3.6 Antimicrobial activity of POLE

The rhizome extract of *P. ostruthium* exhibits broad antibacterial activities (Venugopala et al., 2013). Lakshmanan et al. (2024) indicated that imperatorin, found in the rhizome extract of *P. ostruthium*, exhibits antibacterial properties against *E. coli*, *S. aureus*, *Bacillus subtilis*, and *Klebsiella pneumoniae*. However, oxypeucedanin exhibited no antibacterial activity against *E. coli* or *S. aureus* (Gokay et al., 2010). Limited data are available regarding the antimicrobial activity of POLE, necessitating the evaluation of its antimicrobial activity. Table 3 presents the MBC and MFC of POLE against the tested microbial strains. The MBC of POLE for *S. aureus*, *E. coli*, and *P. aeruginosa* ranged from 150 to 300 mg/L, whereas its MFC for *C. albicans* and *A. brasiliensis* ranged from 350 to 400 mg/L. Therefore, POLE obtained through UAE exhibits strong antimicrobial activity. The high TFC of POLE indicates that flavonoids are the primary microbicidal agents, followed by phenolic acids or coumarins (Venugopala et al., 2013; Widelski et al., 2018). As shown in Table 5, POLE is rich in flavonoids. To validate the antimicrobial efficacy of the optimal POLE concentration (400 mg/L) against the tested strains, we measured the antimicrobial efficacy of the self-formulated lotion by using the USP <51> method. On day 14 of treatment, the lotion’s antimicrobial efficacy against five tested microorganisms was 99.90%–99.95%. By day 28, it increased to 99.96%–99.99%. These results met the acceptance criteria, which require a reduction of at least 2.0 log in bacterial load on day 14 of treatment compared with the baseline load, with no increase from days 14–28. For fungi, the criteria stipulate no increase on day 14 or 28 compared with the baseline load (Sutton and Porter, 2002).

**TABLE 3:** Minimum bactericidal concentration (MBC) and minimum fungicidal concentration (MFC) of *Peucedanum ostruthium* leaf extract against test strains.

MBC (mg/L)			MFC (mg/L)	
*S. aureus*	*E. coli*	*P. aeruginosa*	*C. albicans*	*A. brasiliensis*
250 ± 10	300 ± 25	150 ± 30	350 ± 50	400 ± 20

The ultrasound-assisted extraction operating conditions were as follows: extraction solvent, 80% ethanol; extraction temperature, 61.0°C; ultrasonication duration, 35.5 min; liquid-to-solid ratio, 20 mL/g; and ultrasonic power, 249 W.

### 3.7 Anti-inflammatory activity of POLE

**FIGURE 3:**
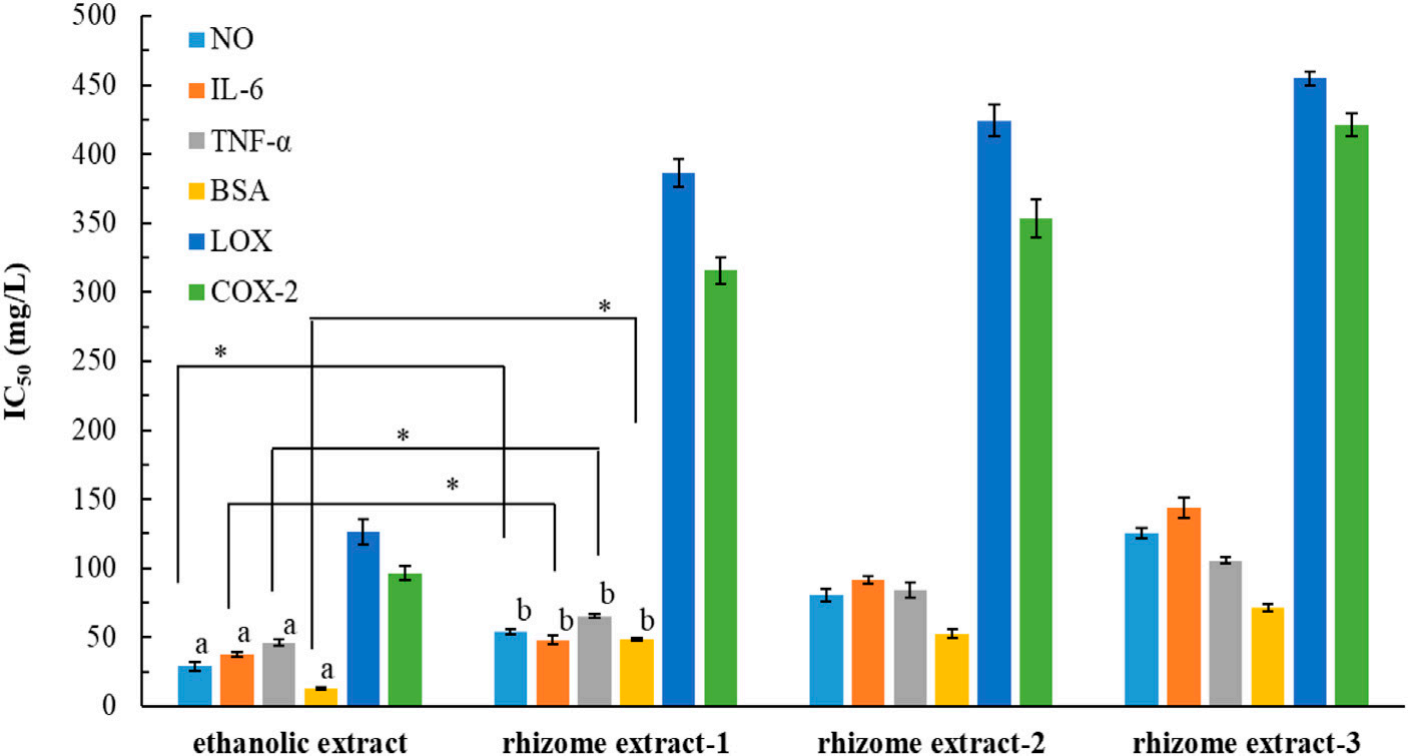
IC_50_ values for *Peucedanum ostruthium* leaf extract and various commercial rhizome extracts against various inflammatory factors. The ultrasound-assisted extraction operating conditions were as follows: extraction solvent, 80% ethanol; extraction temperature, 61.0°C; ultrasonication duration, 35.5 min; liquid-to-solid ratio, 20 mL/g; and ultrasonic power, 249 W. Different letters (a,b) indicate statistically different values according to Duncan’s test at *p* < 0.05.

Hiermann and Schanti (1998) conducted animal studies to investigate the effects of a *P. ostruthium* rhizome extract, which was obtained using 10% ethanol. They found that the extract had anti-inflammatory and antipyretic properties, attributable to coumarins. We evaluated the anti-inflammatory potential of POLE and assessed the possibility of reutilizing waste leaves for various purposes. The IC_50_ values of POLE and the commercial rhizome extracts for different inflammatory factors were determined (Figure 3). The IC_50_ values for POLE ranged from 12.8 ± 0.5 to 126.4 ± 9.4 mg/L, with the extract indicating the highest efficacy in inhibiting BSA activity, followed by NO production (28.9 ± 3.2 mg/L), IL-6 production (37.7 ± 1.6 mg/L), TNF-α expression (46.1 ± 2.3 mg/L), COX-2 activity (96.3 ± 5.2 mg/L), and LOX activity. The anti-inflammatory potential of POLE was 1.3–4.5 times greater than that of the rhizome extracts. When both rhizomes and leaves were extracted using 80% ethanol for 6 h, the leaf extract at 150 mg/L inhibited LOX and COX-2 by 52% and 67.3%, respectively. It exhibited an IC_50_ value of 15.16 mg/L for BSA. By contrast, the rhizome extract inhibited LOX by only 11.3%. It exhibited an IC_50_ value of 57.06 mg/L for BSA (Danna et al., 2022). These findings suggest that UAE not only reduces the extraction time of *P. ostruthium* leaves (to 35.5 min in this study) but also enhances the anti-inflammatory activity of POLE.

### 3.8 Cytotoxicity and wound healing capability of POLE

**FIGURE 4:**
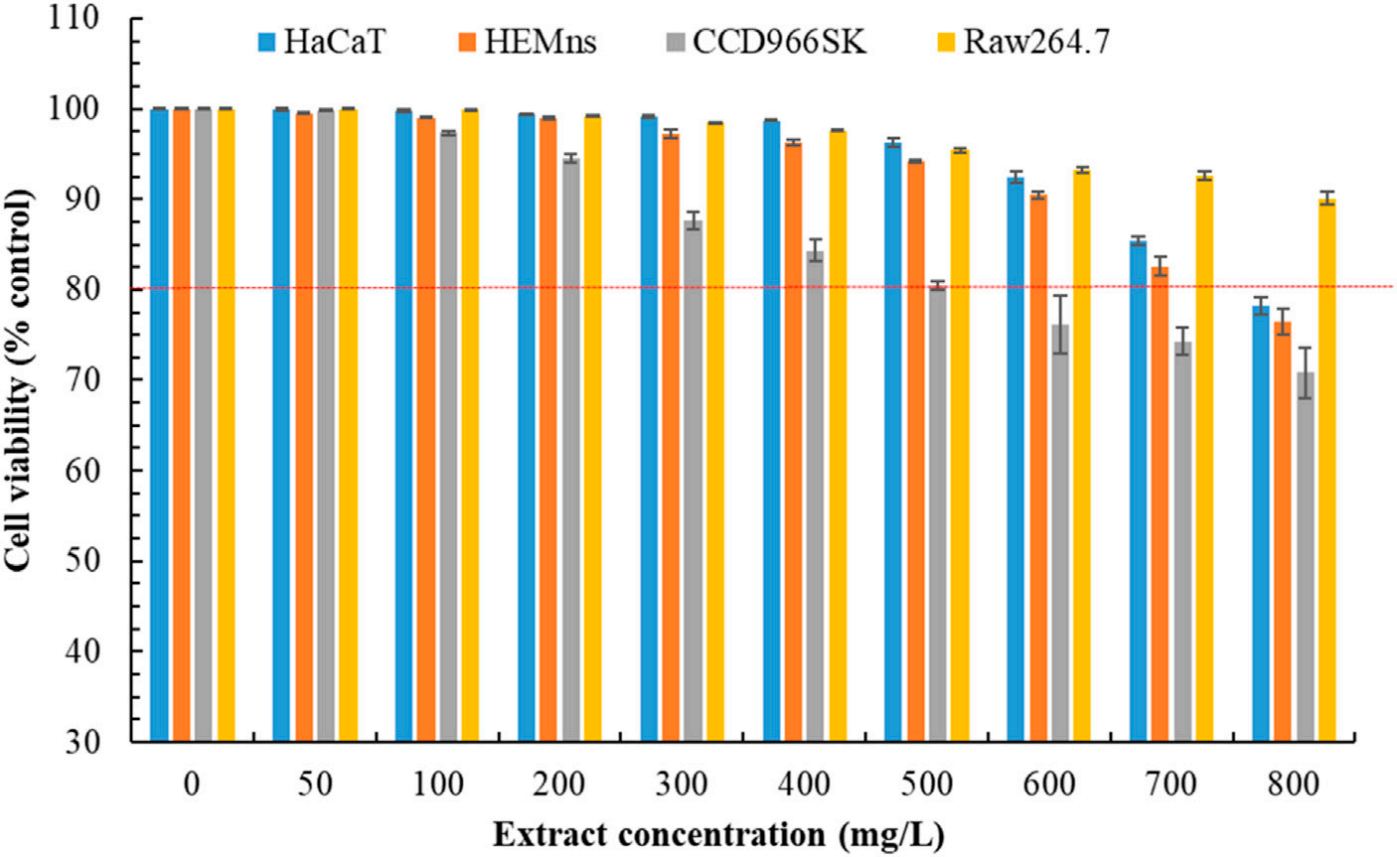
Cytotoxicity effects of *Peucedanum ostruthium* leaf extract at various concentrations on HaCaT, HEMns, CCD966SK and Raw264.7 cells after exposure for 72 h. The ultrasound-assisted extraction operating conditions were as follows: extraction solvent, 80% ethanol; extraction temperature, 61.0°C; ultrasonication duration, 35.5 min; liquid-to-solid ratio, 20 mL/g; and ultrasonic power, 249 W. Data are presented means and standard deviations of three independent experiments.

We previously demonstrated that POLE exhibits strong pharmacological activity. However, assessing its safety is essential. Thus, we evaluated the potential cytotoxicity of POLE on various cell lines. The effects of POLE at various concentrations on cell viability were assessed over a 72-h period (Figure 4). A viability threshold of 80% commonly indicates biological safety (Pintor et al., 2020; Lin et al., 2024). We found that POLE was nontoxic to HEMns and HaCaT cells at concentrations of ≤700 mg/L and to CCD966SK cells at concentrations of ≤500 mg/L. At a concentration of 800 mg/L, POLE exerted no adverse effects on Raw264.7 cells. In summary, POLE obtained through UAE was found to be biologically safe at concentrations of ≤500 mg/L. Thus, this concentration is deemed safe for topical application and potent pharmacological activity. By contrast, the rhizome extract obtained using 80% ethanol exhibited considerable cytotoxicity to HaCaT and L929 cells following 48 h of incubation (Danna et al., 2022). These findings suggest that POLE obtained with 80% ethanol plus UAE exhibits both potent pharmacological activity and biological safety.

**FIGURE 5:**
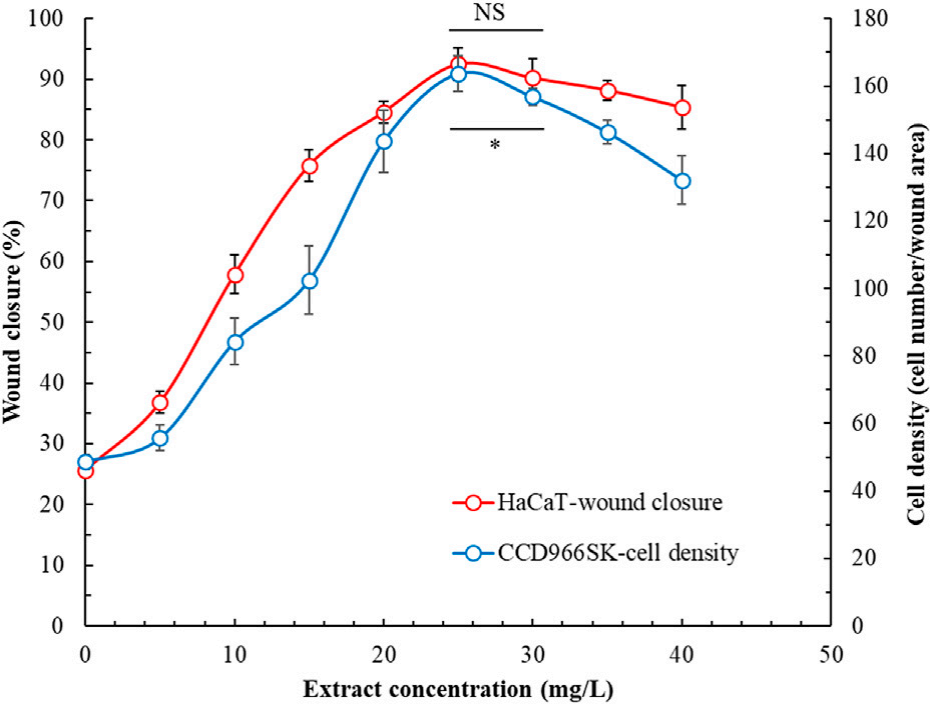
Effects of *Peucedanum ostruthium* leaf extract at various concentrations on the wound healing capability of HaCaT and CCD966SK cells. The ultrasound-assisted extraction operating conditions were as follows: extraction solvent, 80% ethanol; extraction temperature, 61.0°C; ultrasonication duration, 35.5 min; liquid-to-solid ratio, 20 mL/g; and ultrasonic power, 249 W. Data are presented means and standard deviations of 50 wounded cells (NS, nonsignificant; *p* > 0.05, Significance, *p < 0.05).

Figure 5 presents the effects of POLE on the wound healing capacity of HaCaT and CCD966SK cells. With increasing extract concentration, the cell’s wound healing capacity initially improved but then declined. At the optimal concentration of 25 mg/L, the wound healing capacity of HaCaT cells reached 92.5% ± 2.7%, whereas that of CCD966SK cells reached 163.7 ± 5.2 cells/wound area. The rhizome extract and leaf extract of *P. ostruthium*, derived using 80% ethanol, demonstrated wound healing capacities of 30% and 75%, respectively, in HaCaT cells (Danna et al., 2022). The 50 mg/L allantoin (positive control) demonstrated wound healing capacity in HaCaT cells and CCD966SK cells were 81.4% ± 3.2% and 158.1 ± 7.6 cells/wound area, respectively. POLE demonstrated superior wound healing properties. Although POLE exhibited optimal effects at specific concentrations, excessively high concentrations may be detrimental. Therefore, POLE should be used independently to fully harness its pharmacological potential in wound healing.

### 3.9 Major chemical constituents of POLE

Table 4 presents the major volatile organic compounds (VOCs) detected in POLE, including only those with relative contents of >0.5%. A total of 34 predominant VOCs were identified, with caryophyllene oxide (11.24%), α-humulene (11.06%), caryophyllene (7.56%), spathulenol (7.14%), cis-ocimene (5.36%), osthole (5.35%), and cubenol (5.17%) emerging as the most abundant compounds. POLE mainly contained sesquiterpenes (64.11%), monoterpenes (23.80%), coumarins (5.35%), hydrocarbons (4.56%), and diterpenoids (2.18%). Analyses of essential oils extracted (through hydrodistillation) from *P. ostruthium* leaves sourced from Poland and Italy revealed that despite variations in origin and extraction methods, sesquiterpenes remained the dominant class, accounting for 57.8% and 77.1% of the essential oils in Polish and Italian leaves, respectively (Cisowski et al., 2001; Garzoli et al., 2022). Sarkhail (2014) indicated polyphenols, flavonoids, and coumarins as the principal nonvolatile compounds in *Peucedanum*. Thus, we analyzed these compounds through RP-HPLC-ESI-MS in the present study and the MS chromatograms were included in the Supplementary Material. Table 5 presents the major polyphenols and coumarins identified in POLE and corresponding contents. Our analysis unveiled 19 key compounds, including kaempferol 3-O-acetyl-glucoside (30.85%), 4-O-caffeoylquinic acid (10.43%), quercetin-3-O-(6″-acetyl-glucoside) (9.21%), oxypeucedanin (8.81%), quercetin-3-O-rutinoside (8.67%), and oxypeucedanin ethanolate (7.74%). Flavonoids constituted 57.64% of the total composition, followed by coumarins (26.18%) and phenolic acids (16.18%). This distribution aligns with the results of a study exploring the components of POLE obtained using 80% ethanol. However, in the aforementioned study, flavonoids were more dominant (76.23%), whereas coumarins were much less so (6.8%) (Danna et al., 2022). This discrepancy might have influenced the pharmacological activity of the extracts. Coumarins identified in POLE in our study included oxypeucedanin and its derivatives, isoimperatorin, imperatorin, ostruthin, and ostruthol, which were detected also in the rhizome extracts, although at relatively low concentrations (Joa et al., 2011).

**TABLE 4:** The major volatile organic compounds (VOCs) composition and relative content of *Peucedanum ostruthium* leaf extract.

No.	RI*	Chemical Compounds	Categories	Relative content (%)
1	934	α-pinene	Monoterpene	1.25
2	952	camphene	Monoterpene	0.71
3	961	benzaldehyde	Hydrocabons	0.74
4	971	sabinene	Monoterpene	2.93
5	978	β-pinene	Monoterpene	1.85
6	983	myrcene	Monoterpene	2.05
7	1025	*p*-cimene	Hydrocabons	0.61
8	1031	cis-ocimene	Monoterpene	5.36
9	1034	limonene	Monoterpene	0.91
10	1042	1,8-cineole	monoterpene	0.72
11	1062	γ-terpinene	Monoterpene	0.64
12	1098	linalool	Monoterpene	1.02
13	1120	cis-p-mentha-2,8-dien-1-ol	Monoterpene	3.05
14	1178	4-terpineol	Monoterpene	1.51
15	1186	α-terpineol	Monoterpene	1.28
16	1352	α-terpinyl acetate	Monoterpene	0.52
17	1378	α-copaene	Sesquiterpene	0.63
18	1392	β-elemene	Sesquiterpene	1.21
19	1428	caryophyllene	Sesquiterpene	7.56
20	1465	α-humulene	Sesquiterpene	11.06
21	1469	β-humulene	Sesquiterpene	3.24
22	1486	germacrene D	Sesquiterpene	3.25
23	1501	ledene	Sesquiterpene	2.85
24	1507	α-selinene	Sesquiterpene	0.74
25	1509	α-fanesene	Sesquiterpene	2.63
26	1517	valencene	Sesquiterpene	2.75
27	1522	δ-cadinene	Sesquiterpene	3.82
28	1567	nerolidol	Sesquiterpene	0.82
29	1576	spathulenol	Sesquiterpene	7.14
30	1582	caryophyllene oxide	Sesquiterpene	11.24
31	1632	cubenol	Sesquiterpene	5.17
32	1868	diisobutyl phthalate	Hydrocabons	3.21
33	2061	13-epimanool	Diterpenoid	2.18
34	2155	osthole	Coumarin	5.35

The ultrasound-assisted extraction operating conditions were as follows: extraction solvent, 80% ethanol; extraction temperature, 61.0°C; ultrasonication duration, 35.5 min; liquid-to-solid ratio, 20 mL/g; and ultrasonic power, 249 W. *RI, retention indices.

**TABLE 5:** The major polyphenols, flavonoids and coumarins and their relative content of the *Peucedanum ostruthium* leaf extract.

Chemical compounds	Categories	Retention time (min)	Relative content (%)
Gallic acid	Phenolic acid	4.25	2.08
Caffeic acid	Phenolic acid	10.71	0.67
Ferulic acid	Phenolic acid	11.50	0.51
3-O-methylellagic acid	Phenolic acid	15.32	1.24
Rutin	Flavonoids	16.71	2.53
Catechin	Flavonoids	20.40	1.74
4-O-caffeoylquinic acid	Phenolic acid	21.55	10.43
5-O-p-coumaroylquinic acid	Phenolic acid	22.53	1.25
Quercetin-3-O-rutinoside	Flavonoids	23.22	8.67
Kaempferol-3-O-glucoside	Flavonoids	23.75	2.85
Quercetin	Flavonoids	24.80	0.52
Kaempferol	Flavonoids	25.51	1.27
Quercetin-3-O-(6″-acetyl-glucoside)	Flavonoids	26.53	9.21
Kaempferol 3-O-acetyl-glucoside	Flavonoids	27.62	30.85
Oxypeucedanin	Coumarins	28.53	8.81
Oxypeucedanin ethanolate	Coumarins	29.56	7.74
Isoimperatorin	Coumarins	30.32	2.11
Imperatorin	Coumarins	32.87	2.85
Ostruthin	Coumarins	33.84	4.67

The ultrasound-assisted extraction operating conditions were as follows: extraction solvent, 80% ethanol; extraction temperature, 61.0°C; ultrasonication duration, 35.5 min; liquid-to-solid ratio, 20 mL/g; and ultrasonic power, 249 W.

### 3.10 Molecular docking results and potential inhibitory mechanisms

The antioxidant activity of *P. ostruthium* extract is often attributed to its high polyphenol content (Danna et al., 2022), whereas its anti-inflammatory activity is ascribed to coumarins (Sarkhail, 2014). Furthermore, POLE’s wound healing properties are associated with its flavonoid constituents (Nani et al., 2018). To elucidate the mechanisms underlying the inhibitory effects of POLE, we performed molecular docking analysis, taking into account the high-concentration VOCs (e.g., caryophyllene oxide, α-humulene, caryophyllene, spathulenol, cis-ocimene, osthole, and cubenol), phenolic acids, flavonoids, and coumarins. The following active ingredients were investigated: kaempferol 3-O-acetyl-glucoside, 4-O-caffeoylquinic acid, quercetin-3-O-(6″-acetyl-glucoside), oxypeucedanin, quercetin-3-O-rutinoside, oxypeucedanin ethanolate, ostruthin, isoimperatorin, and imperatorin. The following target enzymes were investigated: tyrosinase, aging-related enzymes, antimicrobial enzymes, and proinflammatory enzymes.

Table 6 presents the results of the molecular docking analysis. The analysis associated POLE’s whitening effect primarily with quercetin-3-O-rutinoside (rutin; binding energy: −127.62 kcal/mol), and 4-O-caffeoylquinic acid (binding energy: −126.39 kcal/mol). The analysis revealed four categories of antiaging enzymes. Notably, kaempferol 3-O-acetyl-glucoside markedly inhibited the activities of elastase and hyaluronidase (binding energy: −123.77 and −126.11 kcal/mol, respectively). Quercetin-3-O-(6″-acetyl-glucoside) primarily inhibited the activities of MMP-1 and collagenase (binding energy: −140.28 and −135.29 kcal/mol, respectively). Regarding antimicrobial activity, the inhibition of TyrRS and CYP51 was mainly ascribed to quercetin-3-O-rutinoside (binding energy: −128.16 and −142.85 kcal/mol, respectively) and imperatorin (binding energy: −129.21 and −145.44 kcal/mol, respectively). Regarding anti-inflammatory activity, the inhibition of LOX and COX-2 was mainly attributed to oxypeucedanin (binding energy: −125.16 and −131.12 kcal/mol, respectively).

**TABLE 6:** Results of molecular docking analysis of tyrosinase, MMP-1, elastase, collagenase, hyaluronidase, TyrRS, CYP51, LOX, and COX-2.

Chemical compounds	Tyrosinase	MMP-1	Elastase	Collagenase	Hyaluronidase	TyrRS	CYP51	LOX	COX-2
Caryophyllene oxide	−74.20	−63.50	−72.77	−73.16	−78.31	−65.70	−70.35	−70.09	−71.94
α-humulene	−74.31	−66.09	−69.02	−56.56	−63.83	−63.56	−66.33	−61.90	−66.12
Caryophyllene	−70.49	−63.60	−61.51	−62.32	−63.68	−59.02	−62.99	−57.78	−68.50
Spathulenol	−84.32	−69.90	−66.04	−72.26	−65.67	−64.94	−71.75	−67.25	−79.86
Cis-ocimene	−62.78	−65.02	−63.81	−64.63	−68.66	−62.88	−58.76	−58.58	−66.54
Osthole	−74.38	−85.70	−76.39	−97.36	−82.60	−94.57	−84.53	−89.23	−97.77
Cubenol	−82.27	−75.96	−68.22	−73.74	−81.25	−66.35	−75.24	−64.22	−85.27
Kaempferol 3-O-acetyl-glucoside	−115.85	−124.94	−123.77	−113.19	−126.11	−120.59	−132.97	−124.90	−121.89
4-O-caffeoylquinic acid	−126.39	−118.37	−109.36	−110.31	−108.29	−108.05	−112.86	−122.38	−118.87
Quercetin-3-O-(6″acetyl-glucoside)	−112.61	−140.28	−115.91	−135.29	−120.88	−121.27	−127.51	−110.41	−115.55
Oxypeucedanin	−81.11	−88.15	−76.84	−105.46	−88.59	−100.66	−84.72	−131.12	−125.16
Quercetin-3-O-rutinoside	−127.62	−126.59	−107.51	−115.67	−121.06	−128.16	−142.85	−107.55	−119.07
Oxypeucedanin ethanolate	−93.52	−101.25	−85.43	−112.57	−96.21	−102.56	−90.61	−120.25	−116.35
Ostruthin	−101.42	−91.93	−84.71	−84.85	−93.71	−90.69	−81.55	−91.77	−94.86
Isoimperatorin	−90.67	−92.81	−83.33	−83.40	−93.51	−111.25	−128.50	−99.36	−101.37
Imperatorin	−92.88	−95.37	−80.93	−80.67	−94.13	−129.21	−145.44	−90.76	−104.07

These findings indicate that phenolic acids and flavonoids contribute to POLE’s whitening properties, whereas flavonoids contribute to its antiaging properties. Both flavonoids and coumarins contribute to the antimicrobial and anti-inflammatory properties of POLE. Research suggests that quercetin-3-O-rutinoside derived from the aerial parts of *Hemerocallis* cultivars and 4-O-caffeoylquinic acid derived from the root of *Curculigo latifolia* possess whitening properties (Szewczyk et al., 2020; Nur et al., 2023). Kaempferol 3-O-acetyl-glucoside modulates cellular antioxidants, apoptotic proteins, and inflammatory markers, thereby exerting antiaging effects (Mousa et al., 2019). Quercetin-3-O-(6″-acetyl-glucoside) exhibits strong antioxidant capacity and inhibits enzymes associated with skin disorders (Bhat and Riar, 2017). Imperatorin and quercetin-3-O-rutinoside exhibit potent antibacterial activity against bacteria such as *S. aureus*, *Staphylococcus epidermidis*, *P. aeruginosa*, *B. subtilis*, *K. pneumoniae*, and *E. coli* (Goyal and Verma, 2023; Lakshmanan et al., 2024). Furthermore, oxypeucedanin exerts anti-inflammatory effects; thus, it holds promise for pharmaceutical and cosmeceutical applications (Mottaghipisheh, 2021; Liu et al., 2024).

**FIGURE 6:**
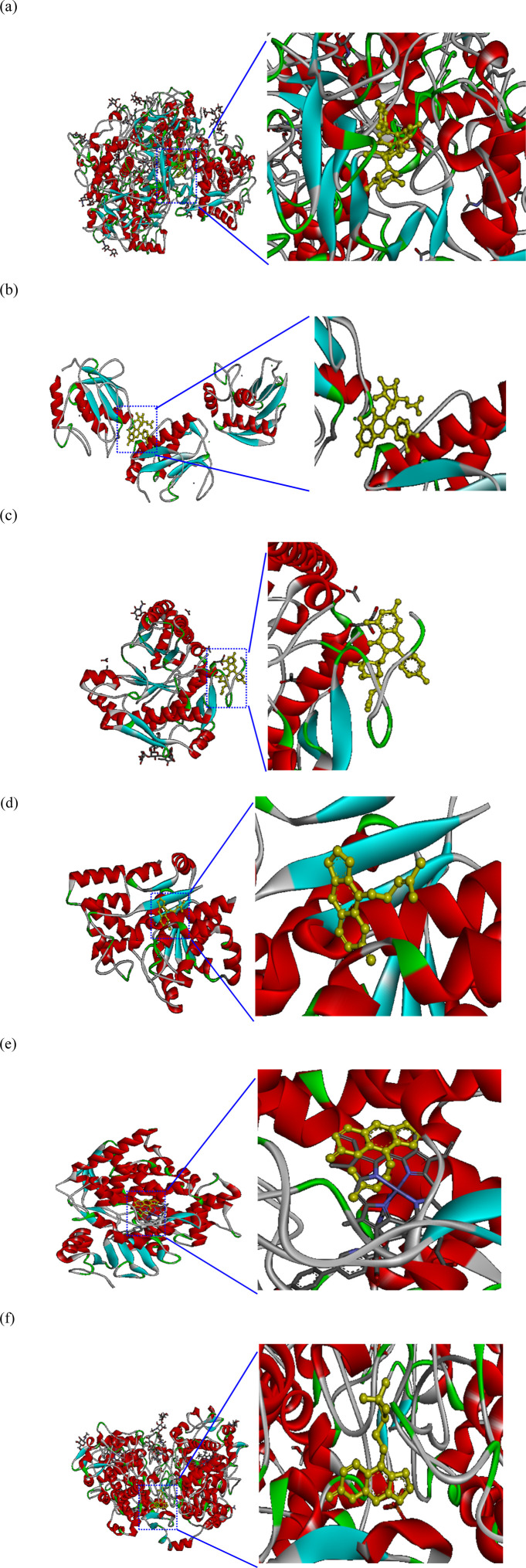
Molecular docking interactions and docking complex of the key inhibitory compounds in *Peucedanum ostruthium* leaf extract against representative enzymes. **(a)** Molecular docking of the interactions between tyrosinase and quercetin-3-O-rutinoside; **(b)** molecular docking of the interactions between MMP-1 and quercetin-3-O-(6″-acetyl-glucoside); **(c)** molecular docking of the interactions between hyaluronidase and kaempferol 3-O-acetyl-glucoside; **(d)** molecular docking of the interactions between TyrRS and imperatorin; **(e)** molecular docking of the interactions between CYP51 and imperatorin; **(f)** molecular docking of the interactions between COX-2 and oxypeucedanin.

Figure 6 depicts the 3D molecular docking results for representative enzymes and key inhibitory compounds in POLE. The compounds exhibited a strong binding affinity to the active sites of the enzymes. Supplementary Figure S2 shows the 2D molecular docking interactions of the key inhibitory compounds in POLE with the representative enzymes. Among the various compounds examined, quercetin-3-O-rutinoside demonstrated the most favorable docking interactions with tyrosinase, aided by the formation of a π–carbon bond with Arg64, π–alkyl interactions with Arg64 and Cys65, as well as H-bonds with Arg64, Glu66, Ala67, His100, Gln437, Asn439, Pro446, and Thr448. Quercetin-3-O-(6″-acetyl-glucoside) exhibited the most favorable docking interactions with MMP-1, facilitated by the formation of a π–alkyl bond with Arg202, a π–sigma bond with Asn143, an amide–π stacked bond with Asp200, an unfavorable acceptor–acceptor bond with Asn143, and H-bonds with Asp124, Asn143, Thr145, Phe149, Arg165, Glu199, and Glu201. Kaempferol 3-O-acetyl-glucoside had optimal docking interactions with hyaluronidase, facilitated by the formation of a π–sulfur bond with Met434, and H-bonds with Asn280, Cys420, Tyr421, Gly423, and Trp424. Imperatorin demonstrated the most favorable docking interactions with TyrRS, facilitated by the establishment of alkyl or π–alkyl bonds with Tyr147, Ala150, and Lys151, a π–π T-shaped bond with Trp196, and H-bonds with Glu152, and Ser153. Furthermore, imperatorin demonstrated the most favorable docking interactions with CYP51, due to a π–sulfur bond with Cys470, alkyl or π–alkyl bonds with Pro375, Phe463, Cys470, and Ala476, a π–sigma bond with Ala476, and H-bonds with Thr311, Cys470, and Gly472. Oxypeucedanin exhibited the best docking interactions with COX-2, facilitated by the formation of alkyl or π–alkyl bonds with Cys36, Cys47, Pro153, Pro154, and Pro156, along with various types of H-bonds with Asn34, Met48, Ser49, Pro154, and Pro156. These interactions contribute to the effective inhibition of enzymatic activity, exerting whitening, antiaging, anti-inflammatory, and antimicrobial effects (Lin et al., 2022). Further research is required to explore the specific bonding mechanisms and underlying biochemical pathways.

### 3.11 Transdermal absorption

On the basis of a prior evaluation of pharmacological activity and cytotoxicity, the optimal concentration of POLE was found to be 400 mg/L. The VOCs in POLE had relatively low molecular weights, with the highest being 290.5 g/mol for 13-epimanool. By contrast, the polyphenolic constituents, particularly flavonoids, had higher molecular weights, with quercetin-3-O-rutinoside reaching a maximum of 772.7 g/mol. All constituents had molecular weights below 800 g/mol, indicating high transdermal absorption efficiency. Because POLE is a composite mixture, we used both TPC and TFC as comprehensive indicators of transdermal absorption efficacy. When evaluated using TPC, the extract and the lotion containing the extract exhibited retention efficiencies of 8.3% and 28.4%, respectively, and penetration efficiencies of 90.2% and 52.5%, respectively (Table 7). Consequently, the total efficiency of transdermal absorption and penetration reached 98.5% and 80.9%, respectively. When evaluated using TFC, the extract and the lotion exhibited retention efficiencies of 11.8% and 30.2%, respectively, and penetration efficiencies of 85.7% and 50.3%. Thus, the total efficiency of transdermal absorption and penetration were 97.5% and 80.5%, respectively. The efficiency of flavonoids was slightly lower than that of polyphenols, likely attributable to the large molecular structures (Bos and Meinardi, 2000). The lotion’s average efficiency of transdermal absorption and penetration was 80.7%, which is markedly lower than that of POLE (98%). This difference may be attributable to the interactions between the compounds in POLE and the lotion matrix (Khan et al., 2015). Nevertheless, the lotion’s observed efficiency aligns well with commercial requirements (Sabbagh and Kim, 2022). These findings support the suitability of POLE for transdermal delivery systems.

**TABLE 7:** Transdermal absorption analysis of the *Peucedanum ostruthium* leaf extract and the lotion containing the extract.

Amount recovery (% of applied dose)					
		Donor	Strat-M	Receptor	Total
TPC	Extracts/lotion	1.2 ± 0.3/18.6 ± 0.7^a^	8.3 ± 0.6/28.4 ± 1.1^a^	90.2 ± 2.0^a^/52.5 ± 1.1^a^	99.7/99.5
TFC	Extracts/lotion	2.3 ± 0.5/19.4 ± 0.8^a^	11.8 ± 0.7/30.2 ± 0.9^b^	85.7 ± 1.8^b^/50.3 ± 0.9^b^	99.8/99.9
Average	Extracts/lotion	1.75 ± 0.4/19.0 ± 0.7	10.05 ± 0.6/29.3 ± 1.0	87.95 ± 1.9/51.4 ± 1.0	99.75/99.7

The ultrasound-assisted extraction operating conditions were as follows: extraction solvent, 80% ethanol; extraction temperature, 61.0°C; ultrasonication duration, 35.5 min; liquid-to-solid ratio, 20 mL/g; and ultrasonic power, 249 W. Within each column, different superscript letters (a,b) indicate statistically different values according to Duncan’s test at *p* < 0.05.

## 4 Conclusion

In the past, the leaves of *P. ostruthium* are typically regarded as waste. Our findings suggest that POLE, which is obtained from discarded *P. ostruthium* leaves through ultrasound-assisted ethanolic extraction, holds promise as a valuable ingredient for skincare and medicinal products. Under the optimal extraction conditions used in this study, POLE exhibited higher TFC and greater pharmacological efficacy than did commercially available rhizome extracts. To the best of our knowledge, this study is the first paper to elucidate various pharmacological activities of POLE—for example, skin whitening, antiaging, antimicrobial, anti-inflammatory, and cellular repair activities. Notably, POLE exhibited low cytotoxicity and favorable transdermal absorption efficiency. Its whitening effect is primarily attributed to phenolic acids and flavonoids, whereas its antiaging effect is attributed to flavonoids. Both flavonoids and coumarins contributed to the antimicrobial and anti-inflammatory effects of POLE. Our findings indicate that the repurposing of these leaves offers a sustainable and cost-effective alternative to the commonly used rhizome in skincare and medicinal formulations. The extensive pharmacological activity of POLE suggests its potential for application in cosmeceutical and healthcare products. Nonetheless, further research is necessary to determine the suitability of POLE for clinical and pharmaceutical use.

## Data Availability

The original contributions presented in the study are included in the article/Supplementary Material, further inquiries can be directed to the corresponding author.
