# Supplementary material for: Ultrasound-assisted extraction of Peucedanum ostruthium leaves: a feasible alternative to rhizomes for industrial applications
Source: Front Pharmacol. 2025 Aug 12;16:1636312. doi: 10.3389/fphar.2025.1636312 (PMC12378154; doi:10.3389/fphar.2025.1636312)
Supplement: Supplementary file 1 [file DataSheet1.docx]

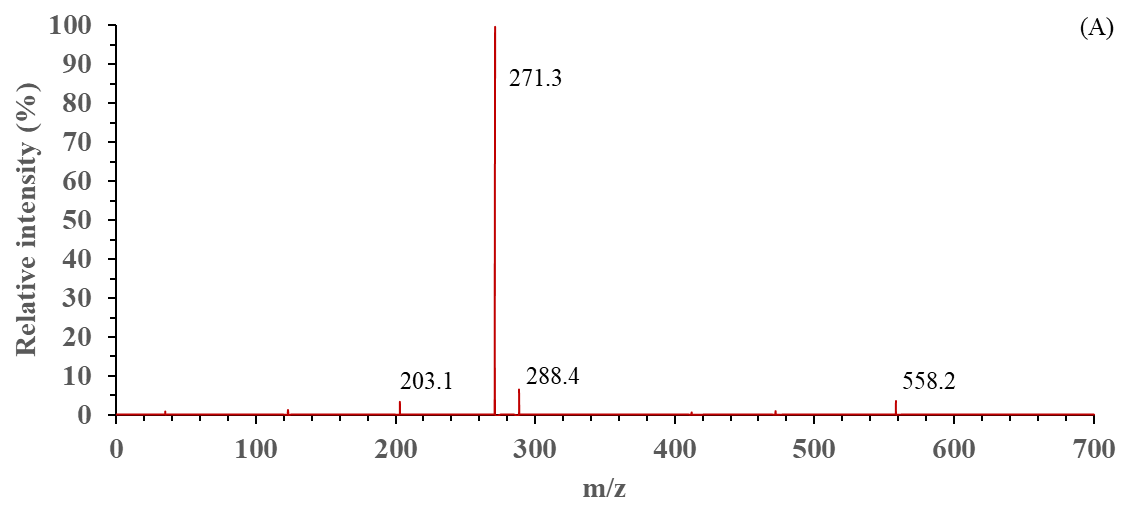


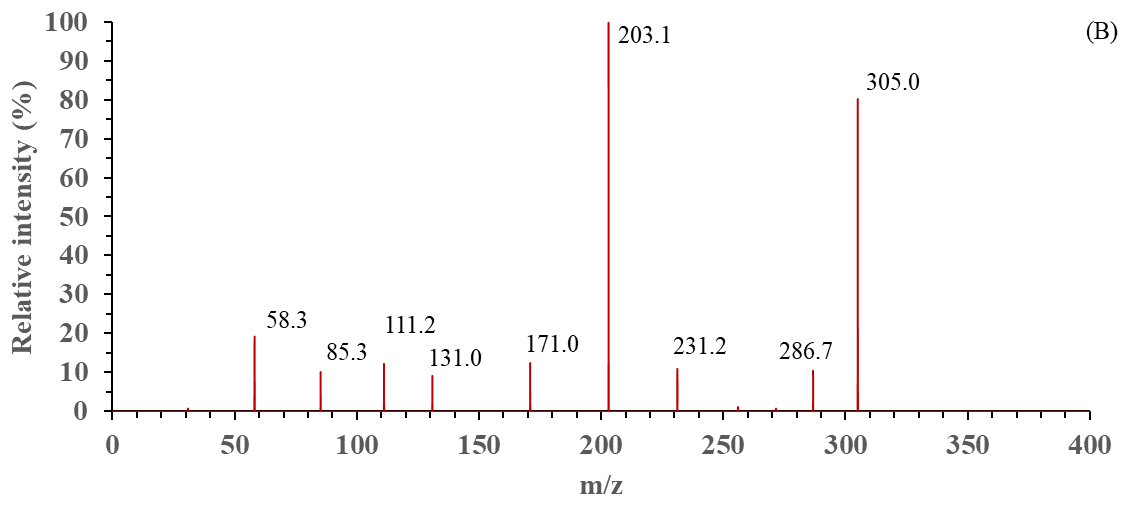


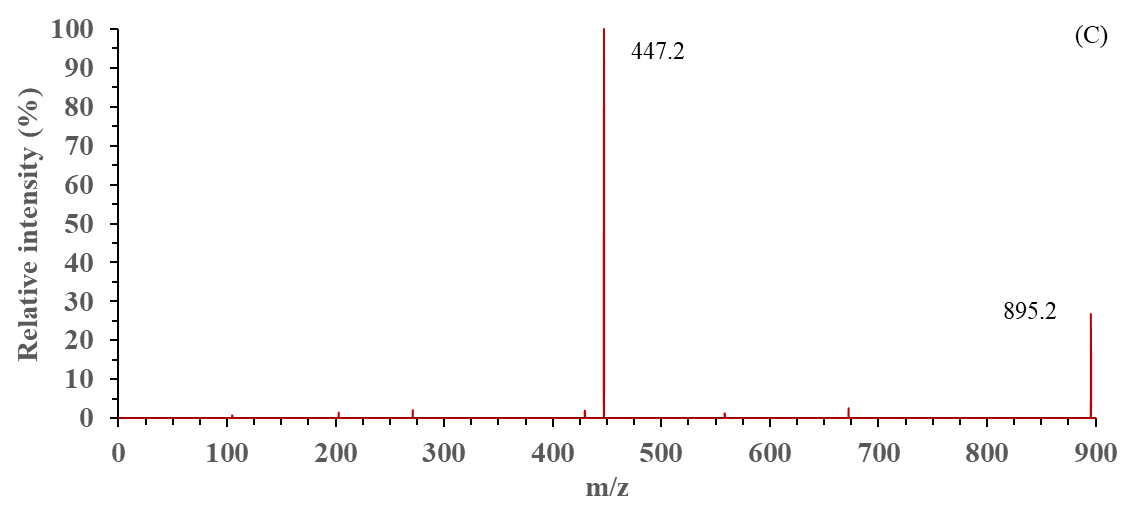


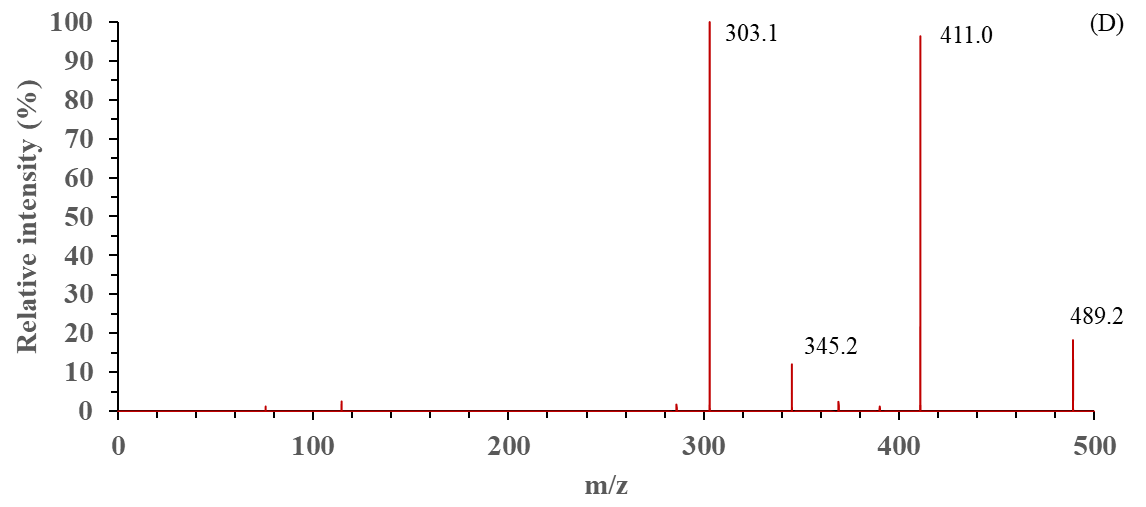


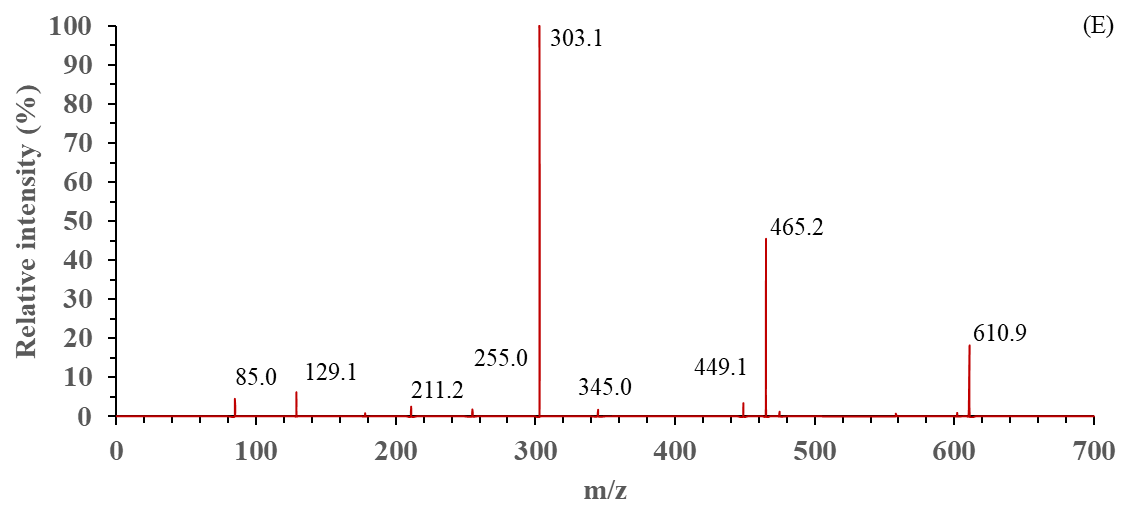


Figure S1. Mass spectra of the major active constituents of *P. ostruthium* leaf extract: (A) imperatorin, (B) oxypeucedanin, (C) kaempferol 3-O-acetyl-glucoside, (D) quercetin-3-O-(6''-acetyl-glucoside), and (E) quercetin-3-O-rutinoside.

| (A)  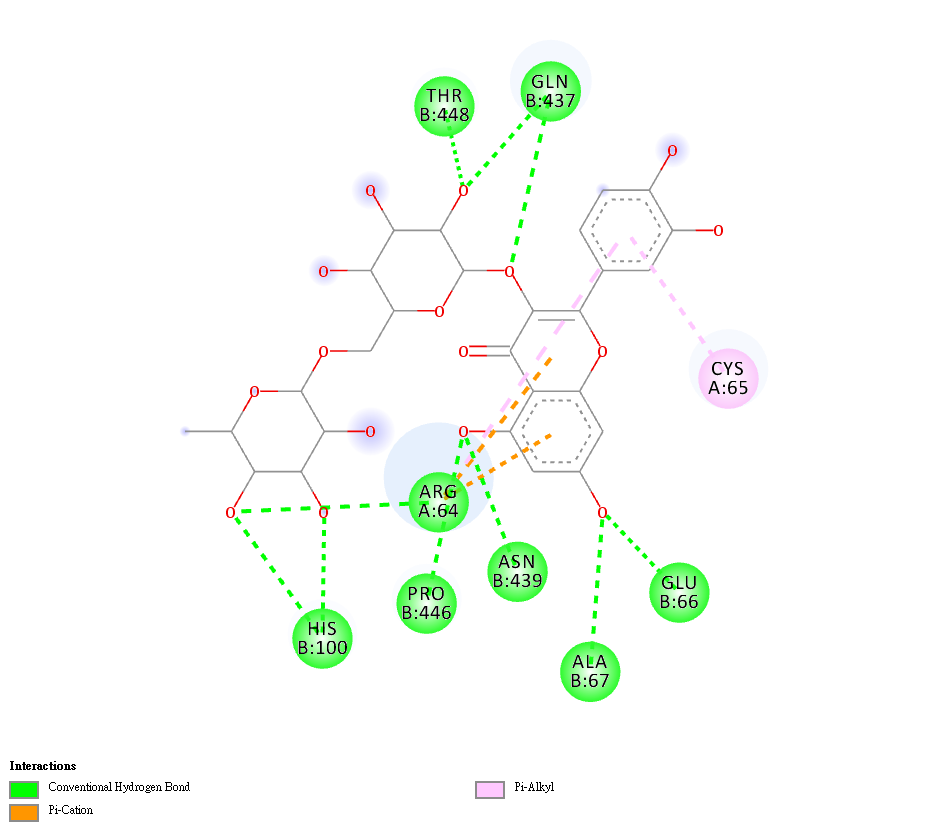 |
| --- |
| (B)  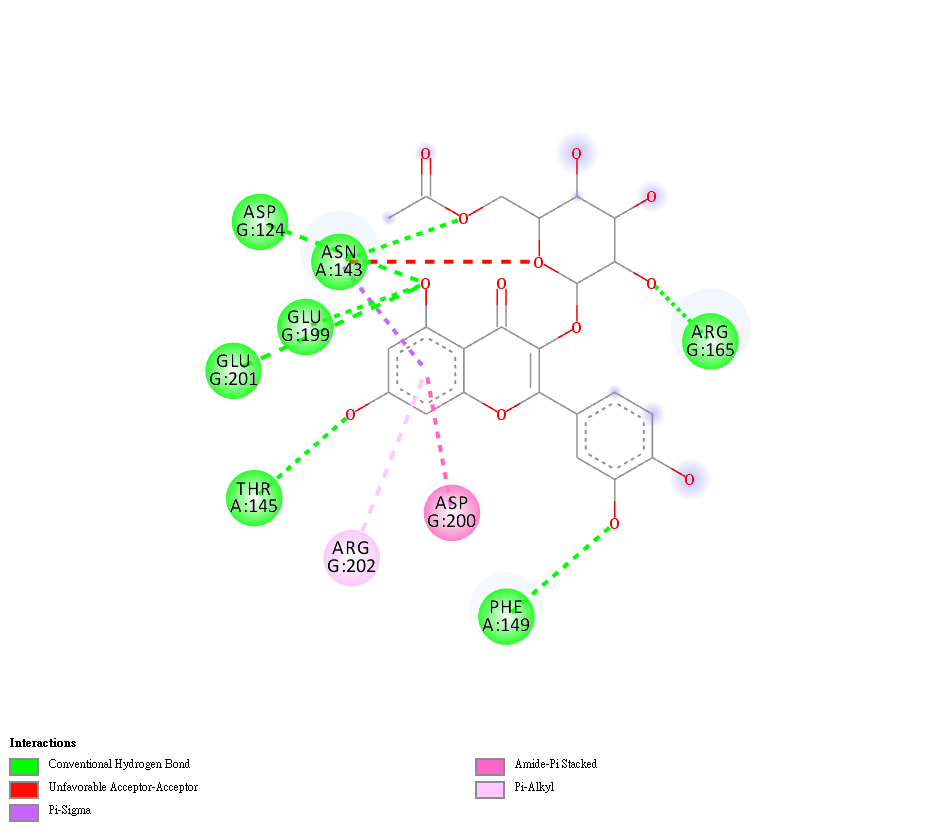 |

| (C)  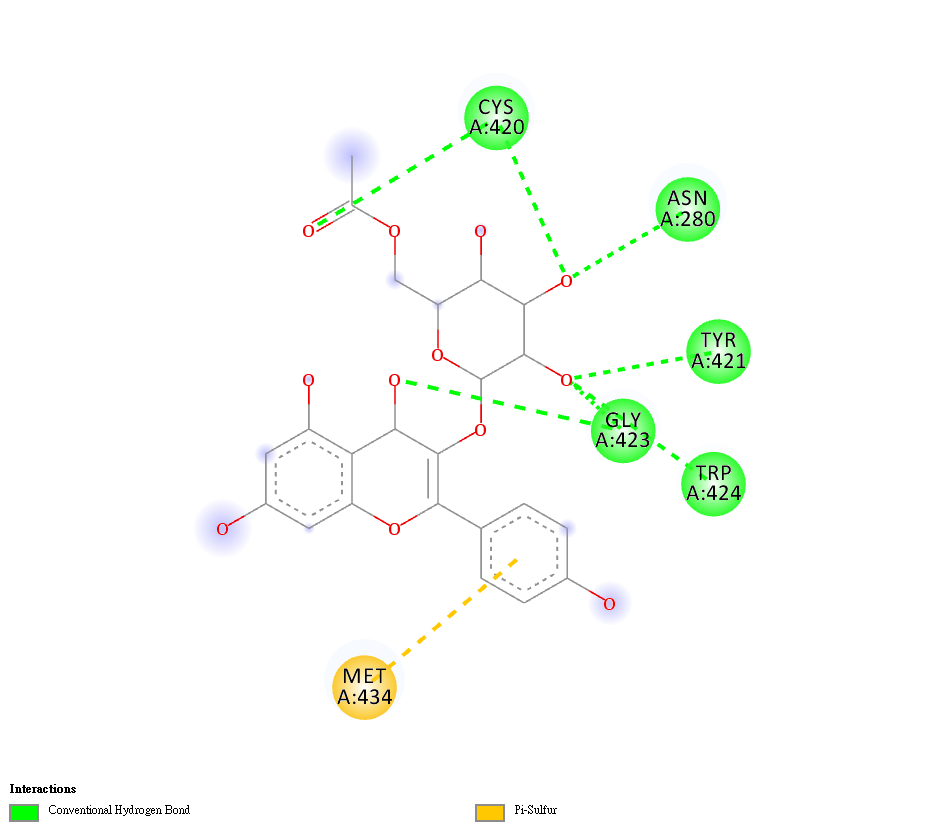 |
| --- |
| (D)  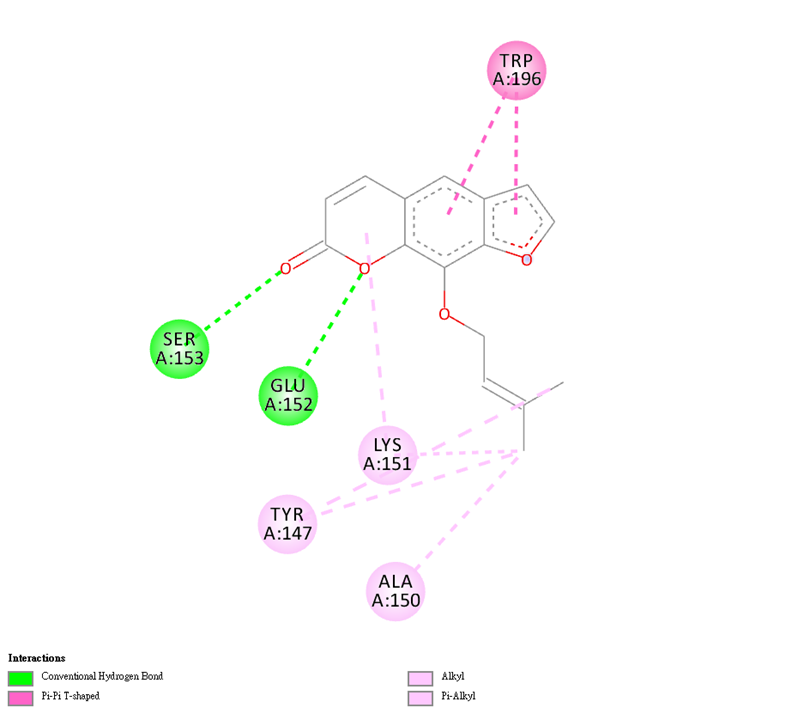 |

| (E)  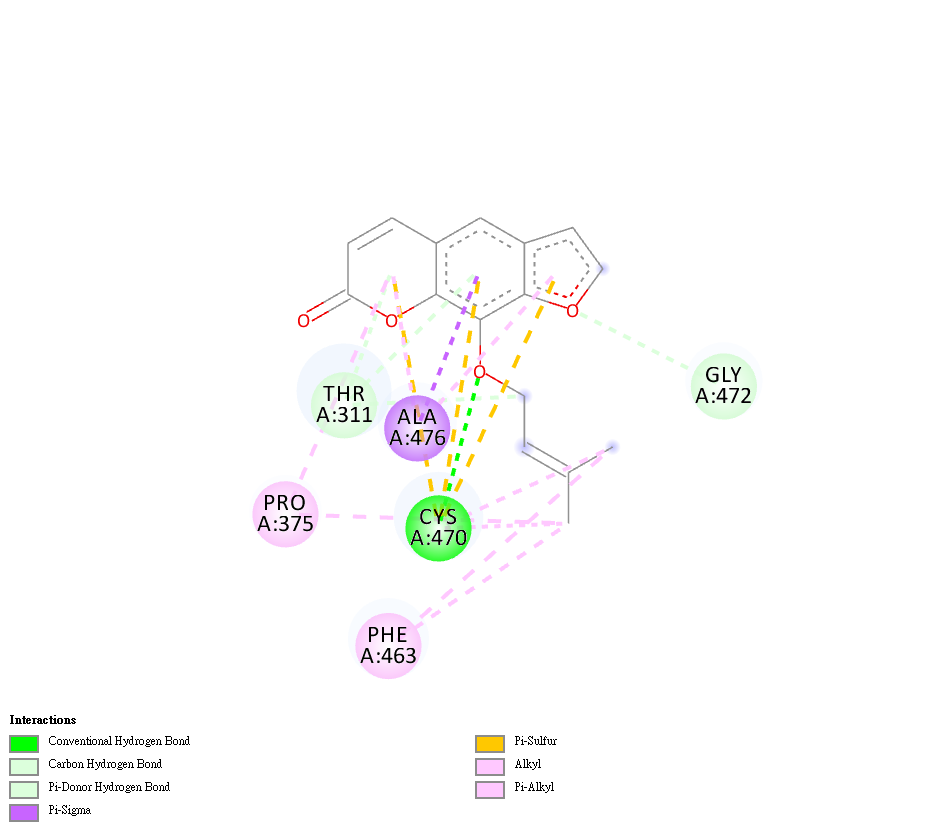 |
| --- |
| (F)  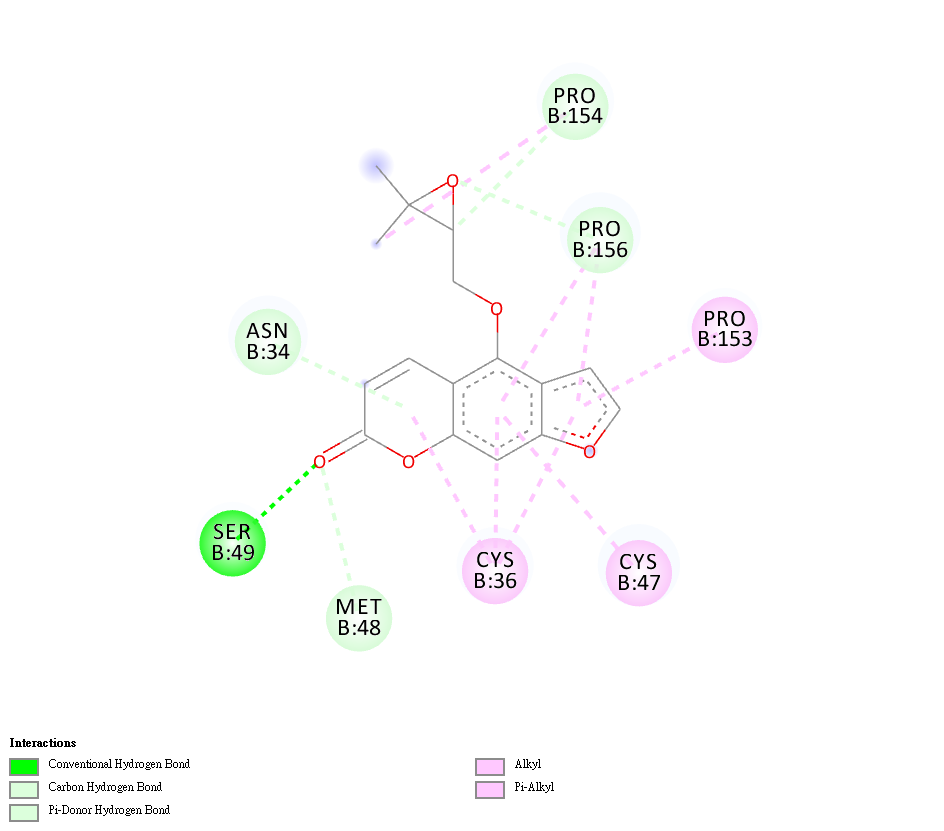 |

Figure S2. 2D molecular docking interactions of the key inhibitory compounds in *Peucedanum ostruthium* leaf extract with representative enzymes. (A) tyrosinase and quercetin-3-O-rutinoside; (B) MMP-1 and quercetin-3-O-(6''-acetyl-glucoside); (C) hyaluronidase and kaempferol 3-O-acetyl-glucoside; (D) TyrRS and imperatorin; (E) CYP51 and imperatorin; (F) COX-2 and oxypeucedanin.
